# Identification of key factors driving inflammation-induced sensitization of muscle sensory neurons

**DOI:** 10.3389/fnins.2023.1147437

**Published:** 2023-05-12

**Authors:** Sridevi Nagaraja, Shivendra G. Tewari, Jaques Reifman

**Affiliations:** ^1^Department of Defense Biotechnology High Performance Computing Software Applications Institute, Telemedicine and Advanced Technology Research Center, U.S. Army Medical Research and Development Command, Fort Detrick, MD, United States; ^2^The Henry M. Jackson Foundation for the Advancement of Military Medicine, Inc., Bethesda, MD, United States

**Keywords:** musculoskeletal pain, nociceptor, ion channels, computational analysis, action potential, sensitization, inflammation

## Abstract

Sensory neurons embedded in muscle tissue that initiate pain sensations, i.e., nociceptors, are temporarily sensitized by inflammatory mediators during musculoskeletal trauma. These neurons transduce peripheral noxious stimuli into an electrical signal [i.e., an action potential (AP)] and, when sensitized, demonstrate lower activation thresholds and a heightened AP response. We still do not understand the relative contributions of the various transmembrane proteins and intracellular signaling processes that drive the inflammation-induced hyperexcitability of nociceptors. In this study, we used computational analysis to identify key proteins that could regulate the inflammation-induced increase in the magnitude of AP firing in mechanosensitive muscle nociceptors. First, we extended a previously validated model of a mechanosensitive mouse muscle nociceptor to incorporate two inflammation-activated G protein-coupled receptor (GPCR) signaling pathways and validated the model simulations of inflammation-induced nociceptor sensitization using literature data. Then, by performing global sensitivity analyses that simulated thousands of inflammation-induced nociceptor sensitization scenarios, we identified three ion channels and four molecular processes (from the 17 modeled transmembrane proteins and 28 intracellular signaling components) as potential regulators of the inflammation-induced increase in AP firing in response to mechanical forces. Moreover, we found that simulating single knockouts of transient receptor potential ankyrin 1 (TRPA1) and reducing the rates of G_αq_-coupled receptor phosphorylation and G_αq_ subunit activation considerably altered the excitability of nociceptors (i.e., each modification increased or decreased the inflammation-induced fold change in the number of triggered APs compared to when all channels were present). These results suggest that altering the expression of TRPA1 or the concentration of intracellular G_αq_ might regulate the inflammation-induced increase in AP response of mechanosensitive muscle nociceptors.

## Introduction

Acute pain is a natural response to muscle injury and is initiated by a specialized class of nociceptive neurons embedded in the muscle tissue. Nociceptors in the muscle respond to noxious stimuli by converting them into electrical signals, i.e., action potentials (APs). Complex signaling among many classes of membrane proteins, such as ion channels, ion pumps, and receptors, contributes to the generation of APs. In addition, nociceptors can become sensitized, i.e., their ability to fire APs (their excitability) can increase, in the presence of inflammatory mediators released by both neurons and non-neuronal cells (e.g., macrophages, neutrophils, and endothelial cells, among others) at the site of tissue injury (Gold and Flake, 2005; Gold and Gebhart, 2010). Nociceptor sensitization is characterized by a reduction in the threshold (stimulus intensity) needed to elicit an AP and an increase in the magnitude (i.e., the number and frequency of APs fired) of its response to a noxious stimulus (Gold and Gebhart, 2010; Waxman and Zamponi, 2014).

**Figure 1:**
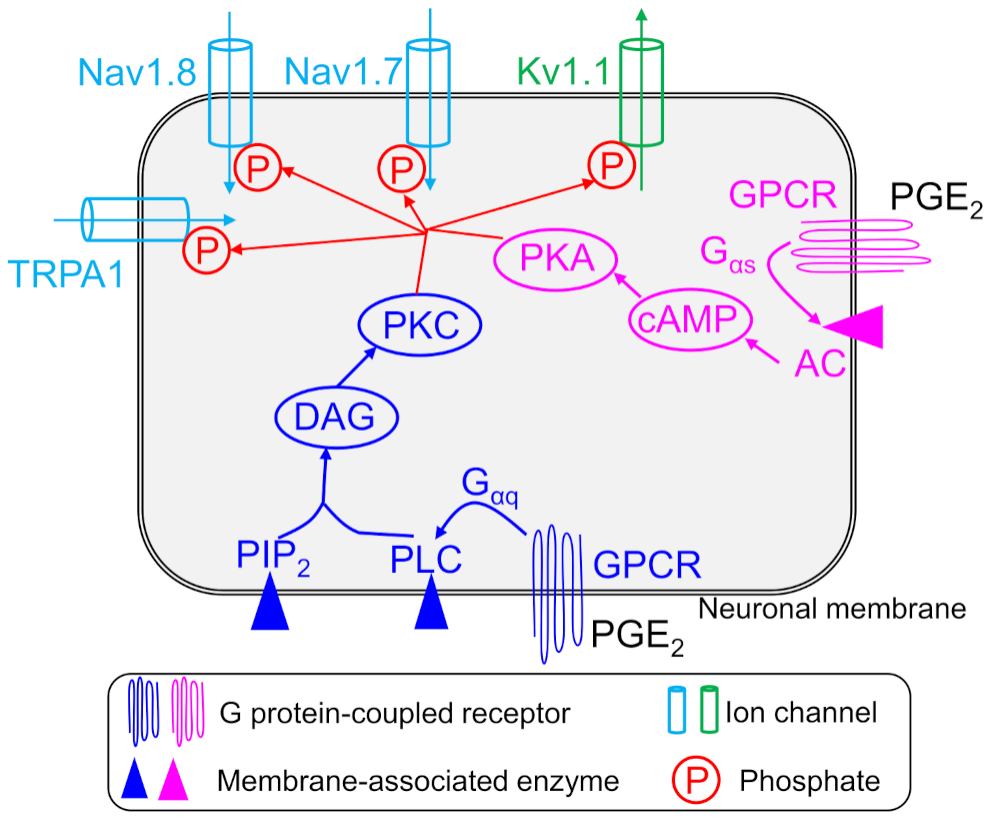
Implementation of inflammation-induced sensitization in a nociceptive afferent neuron model. Shown are the four modeled neuronal transmembrane proteins, TRPA1, Kv1.1, Nav1.8, and Nav1.7, whose activation and inactivation kinetics were modified by intracellular signaling pathways initiated by an inflammatory mediator, e.g., PGE_2_, in the model. The arrows on the ion channels indicate the direction of flow of the ions conducted by those channels. We modeled two pathways initiated by the activation of G protein-coupled receptors (GPCRs) by inflammatory mediators. In the first pathway, phosphorylation of the GPCR activated the G_αq_, β, and γ subunits of the receptor. The G_αq_ subunit then activated membrane-bound phospholipase C (PLC) and phosphatidylinositol 4,5-bisphosphate (PIP_2_) to produce diacylglycerol (DAG). DAG then activated protein kinase C (PKC). In the second pathway, phosphorylation of the GPCR activated the G_αs_, β, and γ subunits of the receptor. The G_αs_ subunit then activated membrane-bound adenyl cyclase (AC) to activate cAMP, which in turn activated protein kinase A (PKA). Finally, PKC and PKA phosphorylated transmembrane proteins TRPA1, Kv1.1, Nav1.8, and Nav1.7 and modified their activation and inactivation kinetics.

We know that numerous inflammatory mediators sensitize muscle afferent neurons, including prostaglandins (PGE_2_), growth factors (e.g., nerve growth factor), cytokines (e.g., IL6, IL1β, and TNFα), neuropeptides (e.g., substance P and bradykinin), lipids, and proteases, among many others (Julius and Basbaum, 2001; Binshtok et al., 2008; Gold and Gebhart, 2010). Many of these mediators activate G protein-coupled receptors (GPCRs) present on afferent neurons, which are capable of coupling to different G protein subunits: G_αq_, G_αs_, or G_αi_. The G_αq_ subunit mediates the activation of phospholipase C-β (PLC-β) and protein kinase C (PKC) (Kuner, 2010), while the G_αs_ subunit is linked to cyclic adenosine monophosphate (cAMP)-protein kinase A (PKA)-mediated sensitization mechanisms (Hucho and Levine, 2007; Gangadharan and Kuner, 2013; Matsuda et al., 2019). Upon activation, PKA and PKC modify the expression and gating properties of various transmembrane ion channels, including Kv1.1, Nav1.7, and Nav1.8, via phosphorylation or other mechanisms, leading to increased neuronal firing (Hucho and Levine, 2007; Gold and Gebhart, 2010) (Figure 1). Typically, neurons return from the sensitized state to their normal excitable state as the injury heals (Gangadharan and Kuner, 2013; Pak et al., 2018). However, in many cases, alterations in the neuronal signaling processes or enhanced expression of certain proteins via transcriptional regulation can cause a long-term increase in the excitability of afferent neurons (Gold and Flake, 2005; Waxman and Zamponi, 2014; Tanaka et al., 2016; Yang et al., 2018). Despite the considerable progress made in identifying the different proteins and processes that participate in inducing neuronal sensitization, we still do not have efficient interventions to prevent the sensitization from becoming persistent because we do not know which specific proteins or processes are key regulators of this event. Thus, identification of such key proteins (or processes) and their associated modifications that lead to persistent neuronal AP firing is essential for improving our understanding of acute pain signaling.

Experimental assessment of muscle nociceptor sensitization is particularly challenging because these neurons are heavily embedded in the muscle tissue, making their nerve endings hard to access. Moreover, compared to other afferent neuron types, muscle nociceptors transduce a variety of noxious stimuli (e.g., thermal, mechanical, and chemical) and exhibit a large diversity in the expression of membrane proteins and firing properties (Mense, 2010). Another factor that complicates *in vivo* investigations of inflammation-induced sensitization is that the production of endogenous inflammatory mediators cannot be precisely monitored, making it a confounding variable in the determination of key contributors to neuronal sensitization. Finally, none of the transmembrane proteins and signaling molecules work in isolation. Therefore, it is important to quantify the contributions of different transmembrane proteins to the AP responses of a sensitized neuron, both individually and relative to the observed changes in the expression and function of other proteins.

Computational modeling can complement traditional experimentation in the search for key proteins or processes that could regulate inflammation-induced sensitization. Using a model, we can compute the effects of knocking out or overexpressing a given protein, or the effects of blocking or activating a molecular process, on AP generation in response to a combination of different types of noxious stimuli and inflammatory mediators in a systematic and time-efficient manner. In fact, previous computational models of pain signaling in nociceptive neurons yielded insights into the roles of specific ion channels. For example, previous models have characterized the contributions of different Na^+^ and K^+^ channels, such as Nav1.6, Nav1.7, Nav1.8, and the Ca^2+^-activated K^+^ channel, to AP generation in neurons innervating the trigeminal nerve (Tanaka et al., 2016), gastrointestinal tract (Chambers et al., 2014), urinary bladder (Mandge and Manchanda, 2018), and other non-specific afferent neuron dorsal root ganglions (Amir and Devor, 2003; Baker, 2005; Tigerholm et al., 2014). However, most of these previous models focused on neurons that did not innervate the muscle tissue and did not incorporate neuronal sensitization by inflammatory mediators. Suleimanova et al. (2020) developed a model to predict the effect of the inflammatory mediators serotonin and adenosine triphosphate (ATP) on the AP firing of afferent neurons in the meninges, a process that leads to migraines, and showed that multiple factors contribute to prolonged AP firing in these neurons during inflammation, including differential activation/inactivation of Nav1.8 channels. However, their model uses a phenomenological representation of the effect of inflammation of neuronal signaling and does not explicitly model any of the intracellular signaling proteins/enzymes activated by those mediators. In addition, due to the high variability exhibited by neurons depending on the physiological tissue they innervate (de Moraes et al., 2017), computational models must incorporate the transmembrane mechanisms that are pertinent to pain signaling in each neuron type. Thus, to understand the inflammation-induced sensitization mechanisms in musculoskeletal tissue, we need to develop a computational model based on experimental data specific to muscle nociceptors and the inflammatory mediators commonly encountered by these neurons.

In this study, we primarily investigated the key transmembrane proteins and intracellular signaling processes that regulate the inflammation-induced sensitization of a mouse muscle nociceptor in response to mechanical forces. To this end, we extended a previously developed and validated mathematical model of a mechanosensitive muscle nociceptor (Nagaraja et al., 2021) to incorporate the kinetics of two major GPCR pathways activated by inflammatory mediators (i.e., PGE_2_ and bradykinin) and the subsequent phosphorylation of four transmembrane proteins (i.e., Nav1.8, Nav1.7, TRPA1, and Kv1.1) by PKC and PKA, which are the final effectors of the two pathways. The current model represents 14 ion channels, two ion pumps, one ion exchanger, four endoplasmic reticulum (ER) membrane proteins, 28 intracellular components (including Ca^2+^ buffering proteins, kinases, enzymes, and second messenger molecules), and 40 associated processes. Upon model validation, we performed a global sensitivity analysis (GSA) by simulating the responses to mechanical forces, first in the absence and then in the presence of an inflammatory mediator, in 50,000 neurons to quantify the contribution of the different modeled proteins and signaling processes to the inflammation-induced change in AP firing magnitude. From this analysis, we identified three ion channels (i.e., TRPA1, Kv7.2, and Piezo2) and four processes (i.e., GPCR phosphorylation, G_αq_ activation, PKA inhibition, and Nav1.8 and Nav1.7 phosphorylation) as key regulators of the inflammation-induced increase in neuronal AP firing. In addition, we investigated the effects of modifying these proteins and processes on the increase in the magnitude of neuronal AP firing, to generate experimentally testable hypotheses regarding the role of these proteins in the inflammation-induced sensitization of mouse muscle nociceptors in response to mechanical stimuli.

## Methods

### Computational model

In this study, we extended our validated computational model of a mouse muscle nociceptor (Nagaraja et al., 2021) to incorporate the sensitization of nociceptors in the presence of an inflammatory mediator. Our previous model, which included 14 ion channels, two pumps, an exchanger, the intracellular concentrations of Na^+^, K^+^, and Ca^2+^, and the membrane potential (*V*_m_), was developed using customized electrophysiological *ex vivo* data collected from mechanosensitive mouse muscle nociceptors and used to simulate responses to a range of mechanical stimuli, from innocuous to noxious (Nagaraja et al., 2021). To that model, we added mathematical descriptions of two GPCR intracellular signaling pathways that are known to be activated in nociceptors by inflammatory mediators, such as PGE_2_. PGE_2_, which is a well-known pain mediator present in inflamed tissues, activates the GPCRs EP1–EP4 and is known to contribute to inflammatory pain in both humans and mice (St-Jacques and Ma, 2014). Specifically, we described the kinetics of two GPCRs, three membrane-associated enzymes, and 14 intracellular proteins and signaling molecules, which are widely regarded as essential molecular mediators of the inflammatory pain response (Julius and Basbaum, 2001; Basbaum et al., 2009; Gangadharan and Kuner, 2013). We describe the two pathways in detail in the Supplementary material.

### Sensitization of the nociceptor

In our implementation of the two GPCR pathways in the model, we assumed that their activation by an inflammatory mediatory led to an increase in the concentrations of PKA and PKC in the mouse muscle neuron. We also used the fact that phosphorylation of ion channels on the neuronal membrane by PKA and PKC typically lowers the threshold (*V*_m_ in voltage-gated channels or mechanical force in mechanosensitive channels) at which they open or close (Vijayaragavan et al., 2004; Gold and Flake, 2005; Gold and Gebhart, 2010). Here, we modeled the effects of PKA and PKC on the gating properties of three voltage-gated channels, i.e., Nav1.7, Nav1.8, and Kv1.1, and one mechanosensitive channel, TRPA1. First, we used two Boltzmann equations (one for PKC and one for PKA) to compute the magnitude of change in *V*_m_ and mechanical force for the gating of the four ion channels, induced by the instantaneous concentrations of these two protein kinases. We initially derived the parameter values for these equations from literature data (Nicol et al., 1997; Wu et al., 2012). Next, to compute the new values of the activation and inactivation thresholds for each of the four channels, we subtracted (for the activation threshold) or added (for the inactivation threshold) the individual changes induced by PKA and PKC from their nominal values. Finally, we used the new activation and inactivation threshold values to compute the change in the currents flowing through the four ion channels [i.e., *I*_Nav1.8_, *I*_Nav1.7_, *I*_Kv1.1_, and *I*_TRPA1_ in Eq. (1)], which ultimately changed the neuronal AP firing. The equations used to describe the nociceptor sensitization are provided in the Supplementary material.

### Model simulations, inputs, and outputs

Our current model of acute inflammatory pain consists of 55 ordinary differential equations (ODEs) and 131 parameters. Each equation represents one model variable, where a variable represents activation or inactivation factors of 17 transmembrane proteins; the intracellular concentrations of K^+^, Na^+^, Ca^2+^, and inositol trisphosphate (IP_3_); the ER Ca^2+^ concentration; the active and inactive subunits of the two GPCRs; three membrane-associated enzymes; concentrations of 12 intracellular proteins and second messenger molecules; and *V*_m_. Supplementary Table S1 provides a list of the model variables, their descriptions, and their initial values. Using a lumped Hodgkin–Huxley-type formalism (Hodgkin and Huxley, 1952), we calculated changes in *V*_m_ at a given time point based on the changes in the currents of all neuronal transmembrane proteins described above as follows:


(1) $$\frac{dV_{m}}{dt}=\left(\begin{array}{l} I_{\mathrm{Nav}1.8}+I_{\mathrm{Nav}1.9}+I_{\mathrm{Nav}1.7}+I_{\mathrm{Piezo}}+I_{\mathrm{ASIC}3}\\ +I_{\mathrm{TRPA}1}+I_{\mathrm{TREK}}+I_{\mathrm{Kv}7.2}+I_{\mathrm{Kv}1.1}+I_{\mathrm{BKCa}}\\ +I_{\mathrm{Ka}}+I_{\mathrm{Kleak}}+I_{\mathrm{CaT}}+I_{\mathrm{CaL}}+I_{\mathrm{PMCA}}+I_{\mathrm{NaK}}\\ +I_{\mathrm{NCX}} \end{array}\right)/C_{m}$$

where *C*_m_ denotes the membrane capacitance and *I* represents the current through the different transmembrane proteins (described by the subscripts). We used 131 parameters to describe all the modeled mechanisms (neuronal and ER membrane as well as those activated by inflammation). Supplementary Table S2 provides a list of the model parameter numbers (used to keep track of the parameters in our simulations), names, values, descriptions, units, and sources of the computational or experimental study from which we adapted or derived their values. We modified a subset of the model parameters (designated as “modified” in Supplementary Table S2) to match the inflammation-induced changes in mechanical threshold from literature data (see “Model calibration and validation” section below). In all simulations, we maintained the extracellular concentrations of K^+^, Na^+^, and Ca^2+^ as well as the volume of the nociceptor nerve ending and its *C*_m_ at constant values. We provide the ODEs and other equations describing all the modeled mechanisms, as well as the Nernst potentials and ionic balances for the intracellular concentrations of Na^+^, K^+^, and Ca^2+^ in the Supplementary material.

To drive the model, we provided as inputs a series of six rectangular pulses with mechanical forces of 0.7, 4, 10, 20, 40, and 100 mN. We applied each pulse for a period of 10 s, with a 20 s delay between pulses, for a duration of 180 s. We applied the first pulse at the 1 h simulation time point. After 47 h of simulation, we provided as input a 30 min rectangular pulse of an inflammatory mediator at a concentration of 1, 10, or 100 nM. Finally, we re-applied the series of six rectangular pulses 30 min after providing the inflammatory mediator. We chose the time point of 30 min to test the inflammation-induced sensitization in the neuron’s response to mechanical forces based on literature data showing that threshold reduction due to inflammation peaked between 30 min and 1 h in rat and mouse neurons (Hendrich et al., 2013). At the end of each simulation, our model generated a 48 h time course for each of the 55 model variables. In all our computational analyses, we focused on the *V*_m_ time course, from which we calculated the total number of APs generated following the application of each pulse of mechanical force as well as the total number of APs fired (obtained by adding the number of APs for each individual force) before and after the addition of the inflammatory mediator. We defined an AP as a *V*_m_ spike of at least 10 mV from its resting value. We used the MATLAB function FINDPEAKS to identify the APs and to record their height and width as well as the simulation time points at which they were generated. Next, we calculated the fold change in AP firing by dividing the total number of APs generated in response to the application of each of the six forces after inflammation by the corresponding value before inflammation. The fold change in AP firing following inflammation calculated using the nominal parameter set represented the baseline inflammation-induced sensitization. In addition, for each concentration of inflammatory mediator, we calculated the percentage reduction in mechanical threshold, i.e., the minimum amount of force needed to elicit an AP at 15, 30, 60, and 90 min after the addition of the inflammatory mediator. We performed all computations in the software suite MATLAB R2015b (MathWorks, Natick, MA) and solved the model equations using the MATLAB solver ODE15s with default tolerance levels.

### Model calibration and validation

#### Model calibration

**Figure 2:**
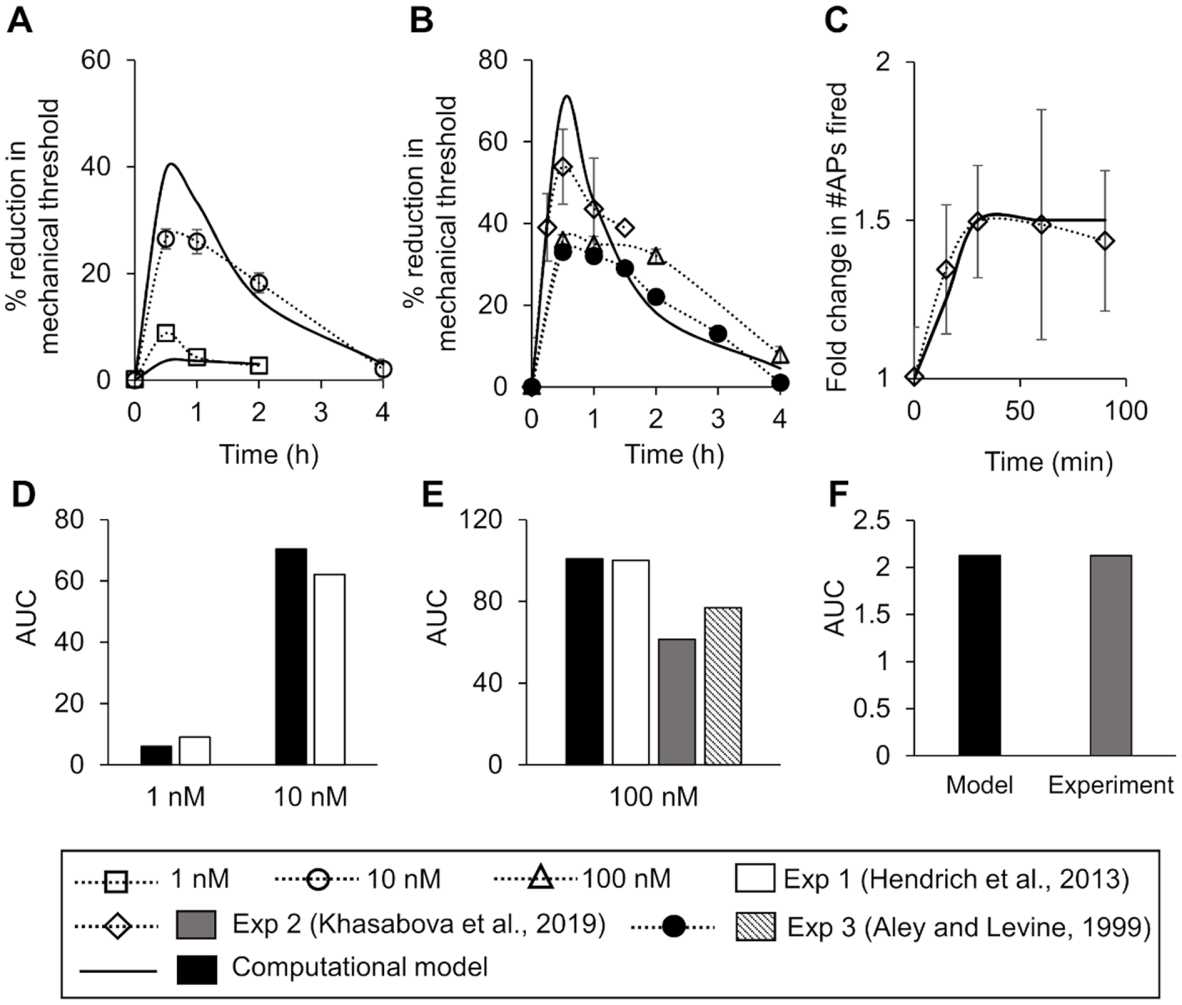
Model calibration and validation. We calibrated the model for mechanical threshold reduction by fitting it to experimental data from rat dorsal root ganglion (DRG) neurons after the addition of two distinct concentrations of an inflammatory mediator. **(A)** Experimental data of the mean percentage reduction in mechanical threshold over a period of 4 h induced by the administration of 10 nM of PGE_2_ (open circles, *N* = 6) and 1 nM (open squares, *N* = 6) of PGE_2_ at time zero (Hendrich et al., 2013). Solid lines show the result of model fitting to the experimental data. We validated the model by comparing the simulations of inflammation-induced mechanical threshold reductions and action potential (AP) firing increase with the corresponding experimental data. **(B)** Experimental data of the mean percentage reduction in the mechanical threshold induced by PGE_2_ administration at 100 mN or higher in rat gastrocnemius muscle neurons (triangles, *N* = 6) (Hendrich et al., 2013), mouse DRG neurons (diamonds, *N* = 10) (Khasabova et al., 2019), and rat hind paw neurons (filled circles, *N* = 12) (Aley and Levine, 1999). Solid line shows the corresponding model prediction. **(C)** Experimental data of the mean increase in the number of APs fired due to inflammation in mouse DRG neurons (diamonds, *N* = 10) (Khasabova et al., 2019). Solid line shows the corresponding model prediction. In all subplots, error bars indicate ±1 standard error of data mean. **(D–F)** Area under the curve (AUC) values for each of the experimentally and computationally derived curves in **(A–C)**, respectively.

To ensure that our model accurately captured the inflammation-induced increase in neuronal excitability (i.e., AP firing) and the reduction in mechanical threshold reported in experiments, we calibrated a subset of 20 of the 131 model parameters to experimental data from the literature. We performed a local sensitivity analysis to determine which model parameters to include in the calibration. To perform the calibration, we modified the values of 20 parameters associated with the kinetics of ion channels Nav1.7, Nav1.8, and Kv1.1, the inactivation of PKC and PKA, and the phosphorylation of Nav1.7, Nav1.8, and TRPA1 (designated as “modified” in Supplementary Table S2) such that the simulated reduction in the mechanical threshold at four time points (i.e., 30 min, 1 h, 2 h, and 4 h) following the addition of 1 and 10 nM concentrations of the inflammatory mediator PGE_2_ matched the corresponding experimental measurements in rat gastrocnemius muscle neurons (Figure 2A) (Hendrich et al., 2013). In addition, to determine the efficacy of the calibration procedure, we calculated and compared the area under the curve (AUC) values for the experimentally and computationally derived curves. We defined the model’s “nominal parameter set” as the final parameter values obtained after performing the calibration procedure.

#### Model validation

To validate our model, we compared its predictions (using the nominal parameter set) to existing literature data we did not use for model calibration. First, we compared its predictions of the reduction in mechanical threshold at four time points (i.e., 30 min, 1 h, 2 h, and 4 h) after the addition of 100 nM of inflammatory mediator to the corresponding data from three different experimental studies performed on neurons derived from mice and rats (Figure 2B). Second, we compared its predictions of the increase in the number of APs in response to a mechanical force of 40 mN after the addition of 100 nM of an inflammatory mediator at 15, 30, 60, and 90 min to the corresponding experimental data from mouse neurons (Figure 2C). In both cases, we also compared the AUC values between the experimentally and computationally derived curves (Figures 2E,F).

### Sensitivity analysis

We performed a sensitivity analysis to identify which proteins and intracellular molecules and their associated signaling processes were key for regulating inflammation-induced changes in neuronal AP firing in response to mechanical forces. First, we performed a local sensitivity analysis (LSA) to assess the model’s robustness and remove any non-essential interactions, as previously described (Nagaraja et al., 2014). In this analysis, we varied the model parameters near their nominal values (±1%). Second, we performed a GSA to account for the known heterogeneity in the expression of the various proteins at different nerve endings of muscle nociceptors as well as the variability in the conductance, activation, and inactivation gating factors of the same membrane proteins under different stimuli (Gold and Gebhart, 2010). For this analysis, we simulated 50,000 distinct nociceptive signaling conditions with inflammation. We first generated 50,000 unique parameter sets by randomly selecting parameter values from a fourfold range (twofold in each direction) around the nominal parameter values. To generate the random parameter sets, we used Latin hypercube sampling (MATLAB function LHSDESIGN) (Nagaraja et al., 2014). Next, we performed simulations using the 50,000 parameter sets, where we drove each simulation using a sequence of six increasing mechanical forces (i.e., 0.7, 4, 10, 20, 40, and 100 mN) applied once before and once 30 min after the addition of an inflammatory mediator. We stopped and eliminated the time course simulations of *V*_m_ that did not reach the 48 h time point within 5 min of computation time (wall-clock) or that required time steps smaller than 1 × 10^−12^ s. We used this lack of convergence in the simulations to flag parameter sets that resulted in non-physiological kinetic behavior. Accordingly, we only used the simulations that ran to completion to calculate the fold change in the total number of APs fired (in response to all six forces) after inflammation. Finally, we separated the simulations into two groups. We defined a group of simulations whose AP fold changes were ≥5 as “sensitized” neurons. We used 5 as the cut-off value for classifying a neuron as “sensitized” because it represented ~60% of the baseline sensitization value (i.e., 8.1), and we wanted to account for the variability (i.e., ~40%) in the levels of inflammation-induced sensitization reported by different experimental studies (Murase et al., 2010; Khasabova et al., 2019). We defined another group of simulations whose AP fold changes were ≤1 as “non-sensitized” neurons because a fold change value ≤1 indicated that the addition of an inflammatory mediator did not increase the AP firing in these neurons. Using these simulation results, we performed two analyses, a partial rank correlation coefficient analysis and a parameter distribution analysis.

#### Partial rank correlation coefficient analysis

For this analysis, we calculated the Spearman’s partial rank correlation coefficient (PRCC) and the associated *p*-values between the primary output (i.e., the AP firing fold change) and each of the 131 model parameter values in the sensitized and non-sensitized neuron groups. The values of the PRCC varied between −1 and + 1, with large absolute values reflecting a high impact of the particular model parameter on the model output (i.e., AP fold change). The sign of the PRCC indicated the positive or negative directionality of the correlation between the model parameter and the output. A PRCC with a *p*-value <0.01 indicated that it was significantly different from zero. Upon completion of this analysis, we obtained two sets of 131 PRCC values, along with their associated *p*-values. We also calculated PRCCs between the AP fold change values for each of the six applied forces and the 131 model parameter values in both neuron groups.

#### Parameter distribution analysis

For this analysis, we first generated histograms of the parameter value distributions for each of the model’s 131 parameters using the MATLAB function HIST, with 50 bins partitioning the interval between the minimal and maximal values for each model parameter in the two groups of simulations (i.e., sensitized and non-sensitized neurons). We calculated the percentage of the simulations for each distribution curve by dividing the number of simulations in which a given parameter’s value fell within the range of a bin by the total number of simulations in that group.

Next, for each model parameter, we quantified the area of overlap between the sensitized and non-sensitized neuron group distributions by calculating the Bhattacharyya coefficient, which varied between 0 and 1, representing no and 100% overlap, respectively, as previously described (Mitrophanov et al., 2015). A small overlap area indicated that a parameter (and the protein it represents) was consistently over (or under) expressed in a sensitized neuron relative to a non-sensitized neuron and was therefore more likely to be associated with inflammation-induced neuronal sensitization than parameters with larger distribution overlap areas.

### Identification of key transmembrane proteins that regulate nociceptor sensitization

We utilized the results from the PRCC and the parameter distribution analyses to identify key transmembrane proteins that could regulate the increase in AP generation in a muscle nociceptor during inflammation. Using the results from the PRCC analysis, we divided the set of 131 PRCCs calculated from both the “sensitized” and “non-sensitized” neuron groups into five clusters using a k-means clustering algorithm (MATLAB function KMEANS) (Nagaraja et al., 2017). We considered the model parameters in the cluster that had the highest absolute PRCC values and also had *p*-values ≤0.01 as key regulators of AP firing during inflammation. Using the results from the parameter distribution analysis, we first ranked the absolute values of the 131 Bhattacharyya coefficients in ascending order and designated the parameters within the top five lowest values as key for AP regulation. Finally, we combined the model parameters identified as key for AP fold change regulation in both analyses and labeled the proteins/molecules or the intracellular signaling processes represented by those parameters as key for AP-response regulation during inflammation.

### *In silico* analysis of modification of model-identified proteins and molecular processes

For each model-identified key transmembrane protein, we performed a set of two simulations in which we either knocked out or overexpressed that protein. To simulate a protein’s knockout, we set the current in Eq. (1) corresponding to that protein to zero, and to simulate a protein’s overexpression, we multiplied the current corresponding to that protein by 2. For each model-identified key molecular process, we also performed a set of two simulations in which we either up- or down-regulated the process. To simulate the up- or down-regulation, we multiplied or divided, respectively, the parameter that represented the rate of the process by 10, unless specified otherwise. First, we performed the simulations with the protein or process modifications described above using a model with the nominal parameter set, which represented an average nociceptive muscle afferent neuron. Then, to verify that we could reproduce the effects of the different modifications in a population of neurons, we repeated the simulations for every modification using 10,000 parameter sets randomly selected from the group of successfully completed simulations in the GSA. Like in the GSA, we stopped the simulations that did not reach the 48 h time point of the *V*_m_ time course within 5 min of computation time (wall-clock) to flag parameter sets where a modification resulted in non-physiological kinetic behavior. In addition, like in the GSA, we separated the simulations that converged successfully into groups of “sensitized” and “non-sensitized” neurons based on the same criteria. Using the simulations that converged successfully in the sensitized neuron group, we calculated the mean ± 1 standard error (SE) of the AP fold change after inflammation, for each of the implemented modifications. Finally, we performed a Wilcoxon rank sum test to compare the mean value of each simulation with a modification to the corresponding simulation without any modification.

## Results

### The model captured inflammation-induced changes in the activation threshold and in the AP firing response to mechanical stimulation

To ensure that the model accurately captured the reduction in mechanical threshold (i.e., the minimum force required to elicit an AP from a neuron) following the administration of an inflammatory mediator, we calibrated the model to data obtained from electrophysiological measurements in rat gastrocnemius muscle neurons (Hendrich et al., 2013). Before the addition of the inflammatory mediator, the minimum force needed to elicit an AP in the model was 0.3 mN. The calibration procedure resulted in the percentage reduction in the simulated mechanical threshold for two different concentrations of the inflammatory mediator (i.e., 1 and 10 nM) to fall within two SE of the data for at least 50% of the time points at which they were measured (Figure 2A) and for the AUC values of the experimental and computational curves to be 9.1 and 6.1, respectively, for 1 nM of inflammatory mediator and 62.2 and 70.4, respectively, for 10 nM of inflammatory mediator (Figure 2D). We designated the final set of model parameter values obtained after this calibration procedure as the nominal parameter set. Next, to assess the stability of the model, we performed a LSA using the nominal parameter set (see section “Sensitivity analysis”) and found that *V*_m_ was not very sensitive (sensitivity indices >100) to any of the model’s 131 parameters, suggesting that the model was stable and robust to small perturbations (±1%) of its nominal values.

To validate the model, we first compared our model predictions (using the nominal parameter set) of the percentage reduction in the mechanical threshold after the addition of 100 nM of an inflammatory mediator to the corresponding data obtained after the administration of 100 nM PGE_2_ from three different experimental studies performed using neurons derived from rat gastrocnemius muscle (Hendrich et al., 2013), rat hind paw muscle (Aley and Levine, 1999), and HbSS-BERK mice (Khasabova et al., 2019). Our predictions were within ±1.96 SE of the validation data for at least 70% of the time points available for comparison (Figure 2B), and the AUC values of the experimental and computational curves were 100.2 (Exp 1), 61.5 (Exp 2), 77.0 (Exp 3), and 100.7 (model), respectively (Figure 2E). Next, we compared our model predictions of the number of APs fired in response to a mechanical force of 40 mN applied at 15, 30, 60, and 90 min after the administration of 100 nM of an inflammatory mediator with corresponding data derived from HbSS-BERK mouse nociceptors in response to PGE_2_ (Figure 2C). The model predictions fell within ±1.96 SE of the data for all of the four time points, and the AUC values for the experimental and computational curves were both 2.1 (Figure 2F). Thus, we developed and validated a computational model of inflammation-induced sensitization in a mechanosensitive mouse muscle nociceptor.

Upon validation, we used the model to establish the baseline inflammation-induced increase in AP firing (see “Model simulations, inputs, and outputs” in Methods). The AP fold change values for the individual forces were 42 for 0.7 mN, 35 for 4 mN, 2 for 10 mN, 8.5 for 20 mN, 6.5 for 40 mN, and 1 for 100 mN. The AP fold change value for the total APs fired was 8.1. We used these values as the baseline sensitization and compared the AP fold change in other simulated scenarios with the baseline values to determine the overall effect of inflammation-induced sensitization in those scenarios.

### Key proteins and processes of inflammation-induced AP response regulation

**Figure 3:**
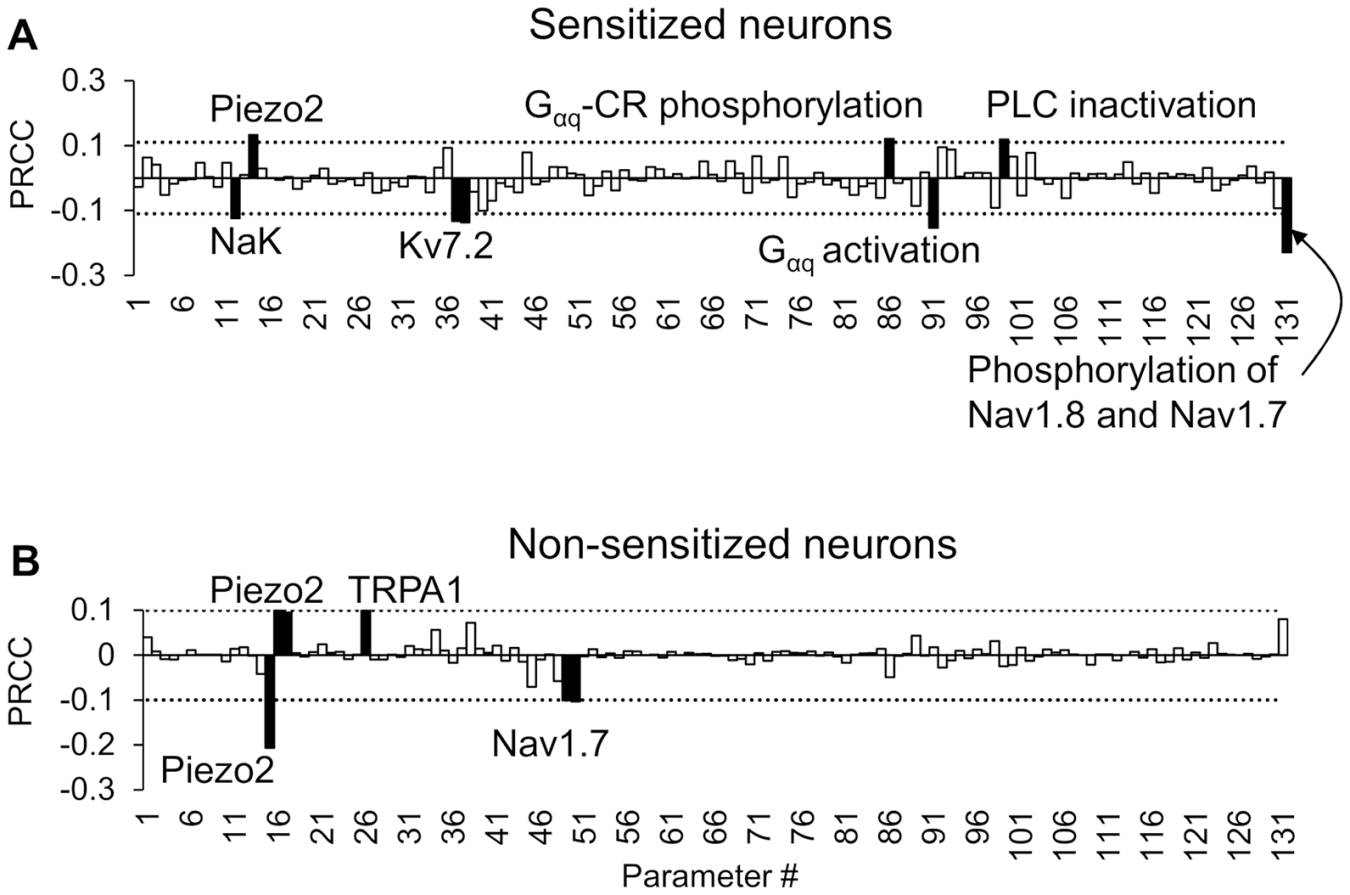
Partial rank correlation coefficient (PRCC) analysis identified key proteins and processes for action potential (AP) regulation. The bars show the PRCCs of the 131 model parameters with fold changes in the total number of APs generated after inflammation calculated from **(A)** 2,042 simulations in which inflammation increased AP firing and **(B)** 14,668 simulations in which inflammation decreased AP firing after the separate application of a series of six mechanical forces of 0.7, 4, 10, 20, 40, and 100 mN. The PRCCs above their respective thresholds (dotted horizontal lines) that were statistically significant (i.e., *p* < 0.01) are indicated by solid black bars, and the labels of the bars show the ion channels/ion pumps or the rates of intracellular processes that these parameters describe in the model. G_αq_-CR: G protein-coupled receptor with the G_αq_ subunit; PLC, phospholipase C.

To identify the transmembrane proteins that strongly regulated the AP response (specifically the number of APs generated) following the addition of an inflammatory mediator across many different nociceptor-signaling conditions, we used two distinct analyses (PRCC and parameter distribution). Of the 50,000 simulations performed for each of these analyses, 48,478 ran successfully. We further classified these simulations into two groups based on the AP fold change values after inflammation (see “Sensitivity Analysis” in Methods). We identified 2,042 simulations as “sensitized” and 14,668 simulations as “non-sensitized” neurons. Using the AP fold change values and the respective parameter values used in each simulation group, we performed the PRCC and parameter distribution analyses. For the sensitized neuron group, the PRCC analysis results showed that the model parameters associated with NaK and Kv7.2 channels yielded high and statistically significant correlations (*p* < 0.01) with the AP fold change values (Figure 3A). In addition, parameters associated with four inflammation-activated molecular processes, namely, G_αq_-coupled receptor phosphorylation, G_αq_ subunit activation, PLC inactivation, and phosphorylation of Nav1.8 and Nav1.7, also yielded high and statistically significant correlations (*p* < 0.01) with the AP fold change values (Figure 3A, solid black bars). For the non-sensitized group, the model parameters associated with Piezo2, TRPA1, and Nav1.7 channels were strongly correlated to the AP fold change (Figure 3B). Not surprisingly, in the non-sensitized neuron simulations, none of the parameters associated with inflammation-activated processes (parameters 88–131) yielded high PRCC values.

We also calculated the PRCC values between the AP fold change values in response to each of the six forces, individually, and the respective parameter values, for both the sensitized and non-sensitized neuron groups (Supplementary Figures S1, S2). In addition to parameters associated with Kv7.2, G_αq_-coupled receptor phosphorylation, and G_αq_ subunit activation, we identified a few other parameters that demonstrated high PRCC values. For example, in the sensitized neuron group, in response to forces of 10 and 20 mN, the parameter associated with PKA activation yielded high and statistically significant correlations (*p* < 0.01) with the AP fold change values (Supplementary Figure S1). In the non-sensitized group, in response to forces of 0.7, 20, and 40 mN, the parameter associated with Kv7.2 activation yielded high and statistically significant correlations (*p* < 0.01) with the AP fold change values (Supplementary Figure S2). However, the sign of the PRCC value (positive or negative) of the Kv7.2-associated parameter was reversed compared to when the same parameter demonstrated high PRCC values in the sensitized neurons, indicating that while Kv7.2 was key in both neuron groups, its expression or activity was altered in an opposite manner.

**Figure 4:**
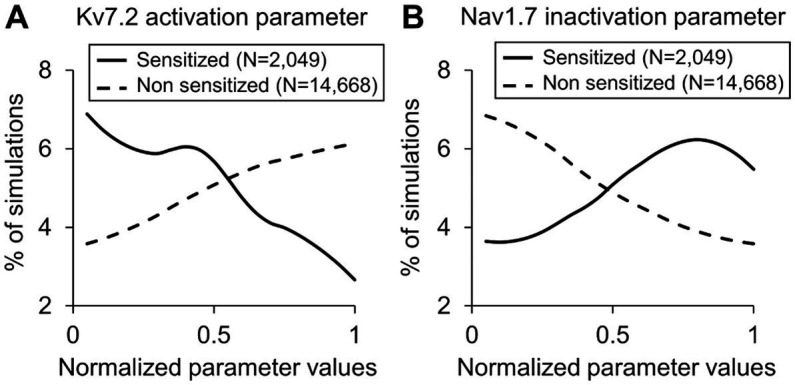
Distributions of parameter values representing **(A)** Kv7.2 activation and **(B)** Nav1.7 inactivation across the simulations of the sensitized neuron group (solid lines) and the non-sensitized neuron group (dashed lines). The *x*-axis indicates the normalized parameter values, and the *y*-axis represents the percentage of simulations in each neuron group in which the parameter values fell within a particular range (described in the “Methods” section).

In the parameter distribution analyses, we calculated the 131 Bhattacharyya coefficients to determine the overlap between the distributions of the normalized values of the model’s 131 parameters in the sensitized and non-sensitized neuron groups. While none of the parameters had considerably low overlap between the two groups of the 131 parameters, the parameters that demonstrated the five lowest values were associated with activation and inactivation of ion channels Nav1.7, Kv7.2, and Piezo. Supplementary Table S3 provides a list of the 131 coefficients for all the model parameters. Figure 4 shows representative examples of two such parameters. For the parameter representing Kv7.2 activation, a larger percentage of the simulations in the sensitized neuron group fell in the lower range of its normalized values compared to those of the non-sensitized group (Figure 4A, solid vs. dashed lines), indicating that Kv7.2 channel expression or its activation might be downregulated in sensitized neurons. Conversely, for the parameter representing Nav1.7 inactivation, a large percentage of the simulations in the sensitized neuron group fell in the higher range of its normalized values compared to those in the non-sensitized neuron group (Figure 4B, solid vs. dashed lines), indicating that the expression or activation of these channels might be downregulated in sensitized neurons. Like for the PRCC analyses, we repeated the parameter distribution overlap calculations for each specific force and found that, overall, the same group of parameters demonstrated lower Bhattacharyya coefficients between their distributions in the two neuron groups for the individual forces (Supplementary Figure S3).

Finally, we combined the results of both analyses (i.e., we added the parameters identified as key in the PRCC analysis to those from the parameter distribution analysis after removing repetitions) and identified three ion channels (Kv7.2, TRPA1, and Piezo2) and four processes (G_αq_ activation, G_αq_-coupled receptor phosphorylation, PKA inhibition, and both Nav1.8 and Nav1.7 phosphorylation) whose modification could potentially alter the inflammation-induced sensitization of mechanosensitive mouse muscle nociceptors.

### *In silico* analysis of the model-identified key proteins and molecular processes

**Figure 5:**
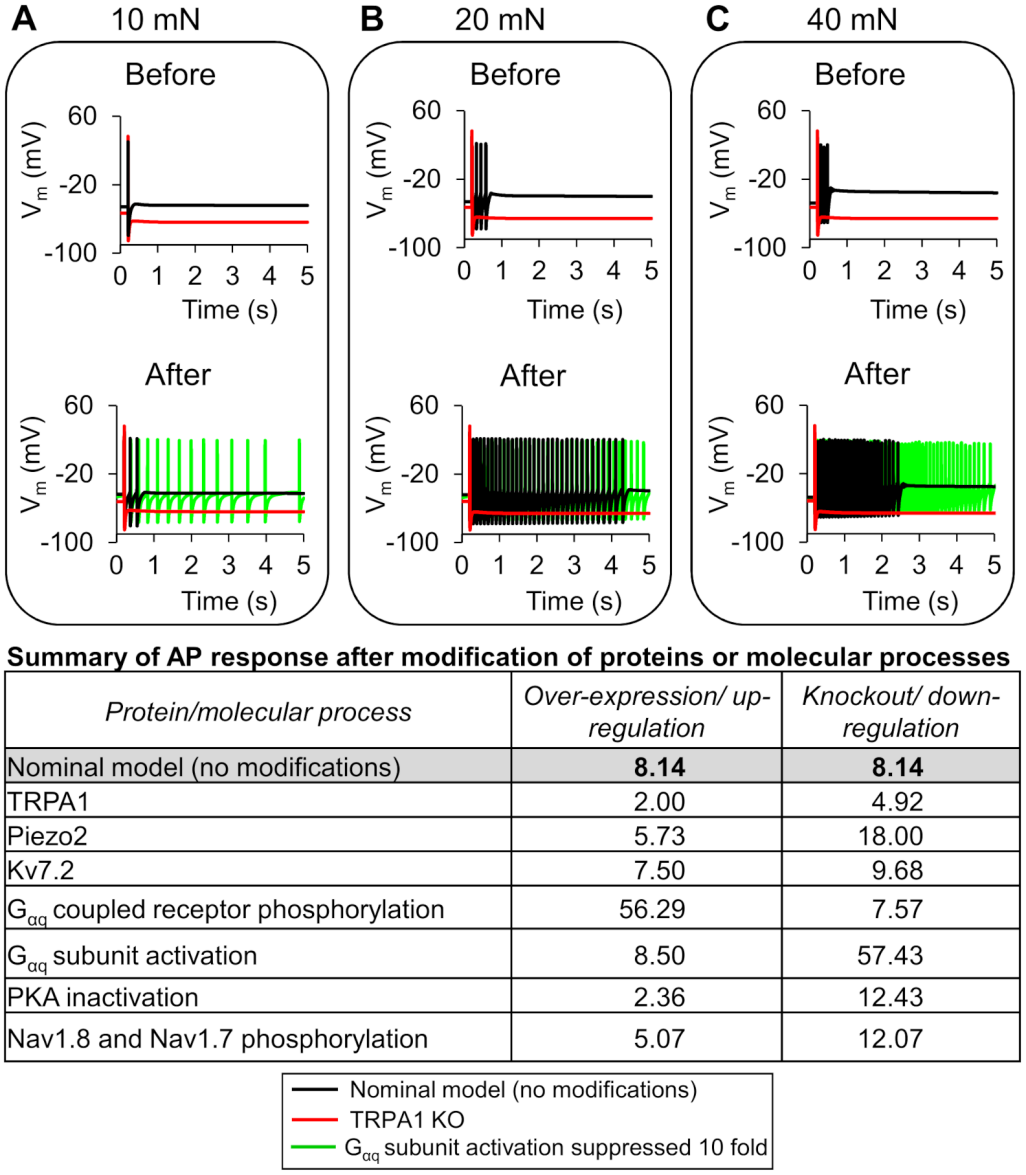
*In silico* analysis identified ion channels and molecular processes that might contribute to inflammation-induced changes in action potential (AP) generation. We simulated the knockout (KO) and two-fold expression increase of three key model-identified ion channels (TRPA1, Piezo2, and Kv7.1) and the increase and decrease in the rates of four key model-identified key processes (GPCR phosphorylation, PKA inhibition, G_αq_ activation, and both Nav1.8 and Nav1.7 phosphorylation) using the nominal parameter set. The figure shows 5 s time courses of the membrane potential (*V*_m_) before and after the addition of an inflammatory mediator, simulated using the nominal parameter set with all channels present (solid black line), with TRPA1 KO (red line), and with the G_αq_ subunit activation rate reduced by 10-fold in response to mechanical forces of **(A)** 10 mN, **(B)** 20 mN, and **(C)** 40 mN. In the top panels of **(A–C)**, which depict the AP response before addition of an inflammatory mediator, the black line representing the *V*_m_ changes in the nominal model (no modifications) overlaps the green line representing the effect of G_αq_ activation reduction. The table shows the magnitude of AP fold change values for every modification performed using the nominal parameter set.

To quantify the effects of each model-identified key protein and molecular process on inflammation-induced sensitization, we performed simulations where we modified a protein or a process, one at a time, using models based on both the nominal parameter set as well as 10,000 parameter sets randomly selected from the group of 48,478 successfully completed simulations in the GSA. Specifically, for the three proteins TRPA1, Piezo2, and Kv7.2, we simulated the effects of their knockout and overexpression on AP firing. For the four processes, we simulated the effects of increasing or decreasing their rates on the AP firing fold change post-inflammation. We then compared the AP fold changes in each of these cases to the corresponding value in the simulation with no modifications (i.e., the baseline sensitization). In the simulations with the nominal parameter set, TRPA1 knockout caused the greatest reduction (Figure 5A, black line vs. red line) in the AP firing fold change in response to 10, 20, and 40 mN forces post-inflammation, whereas a 10-fold reduction in the G_αq_ activation rate caused the greatest increase (Figure 5A, black line vs. green line). The table in Figure 5 shows the AP firing fold change caused by all the modifications compared to the nominal model. In addition, in Supplementary Table S4, we provided the predicted number of APs fired in response to mechanical stimuli before and after the addition of an inflammatory mediator for all the different modifications, along with their fold changes.

**Figure 6:**
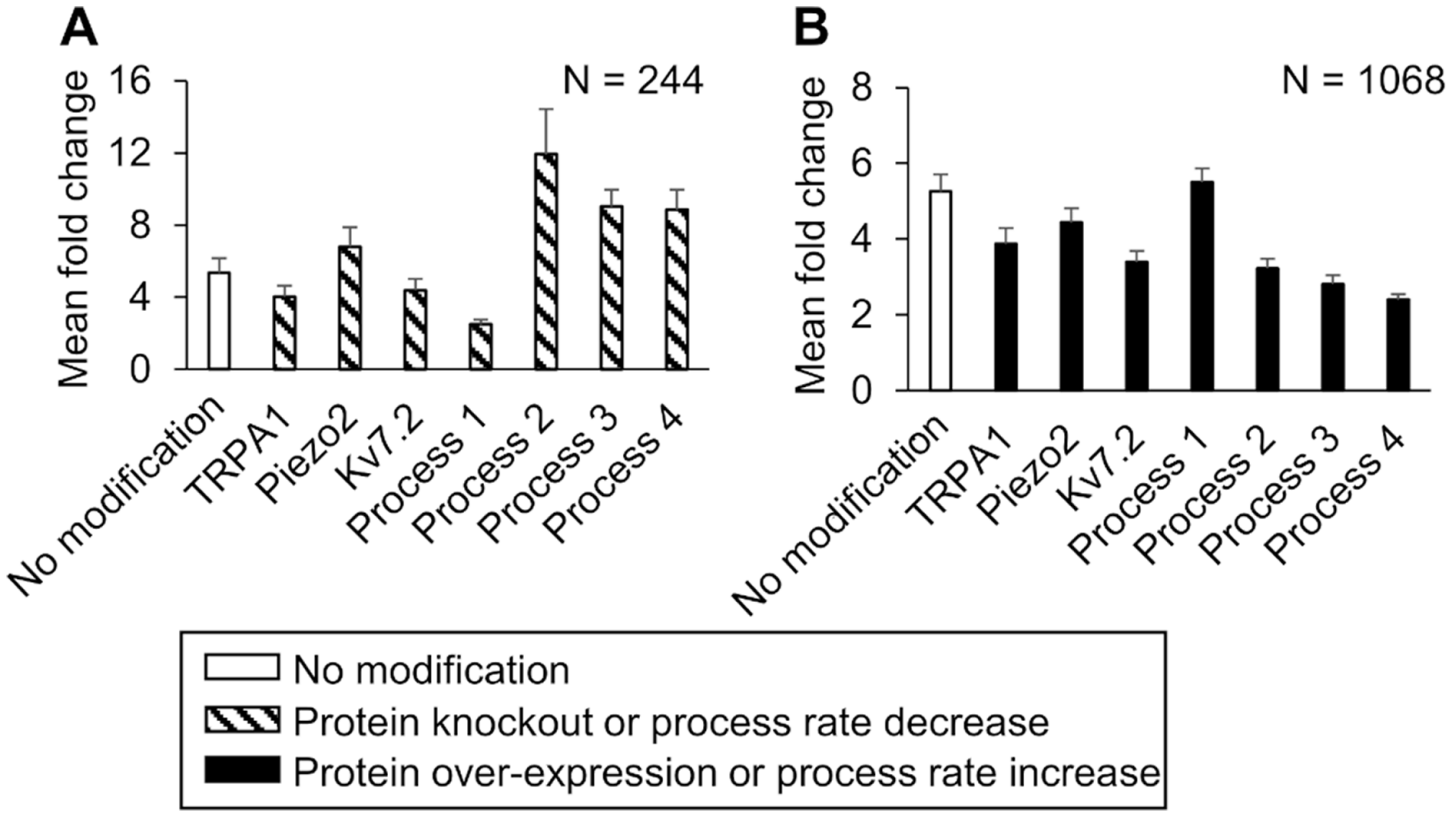
*In silico* analysis identified ion channels and molecular processes that might contribute to inflammation-induced changes in the magnitude of action potential (AP) generation. We simulated either a knockout or a two-fold expression increase of three key model-identified ion channels (TRPA1, Piezo2, Kv7.1) and either an increase or a decrease in the rates of four key model-identified processes (GPCR phosphorylation, PKA inhibition, G_αq_ activation, and both Nav1.8 and Nav1.7 phosphorylation) using 10,000 randomly selected parameter sets. **(A)** Shown are the means and one standard error (SE) of the AP fold change from 244 simulations with seven modifications involving the individual knockout of proteins or 10-fold reduction of the rates of the four key processes (dashed bars). **(B)** Shown are the means and one SE of the AP fold change from 1,068 simulations with seven modifications involving a two-fold expression increase of the key proteins or a 10-fold increase in the rates of the four key processes (solid bars). In **(A)** and **(B)**, the open bar indicates the mean and one SE of the magnitude of AP fold change in simulations with no modification. For implementing the modifications of *process 4*, we increased or decreased its rate by two-fold because the simulations with a 10-fold change did not run successfully. *Process 1:* G protein-coupled receptor phosphorylation, *process 2:* G_αq_ activation, *process 3:* protein kinase A inactivation, and *process 4:* Nav1.8 and Nav1.7 phosphorylation.

In the simulations with the 10,000 unique parameter sets, between 9,021 and 9,663 simulations ran to completion, depending on the implemented modification. Of these, we identified 8,507 simulations that ran successfully for all the implemented modifications. From this group, we further filtered out simulations that represented “sensitized neurons,” i.e., the parameter sets in which the AP firing fold change after simulating a modification was ≥60% of the corresponding value in the simulation with no modification. We found 244 such simulations in the protein knockout and process down-regulation simulation groups, and 1,068 such simulations in the protein over-expression and process up-regulation groups that satisfied the above-mentioned criteria. To determine the magnitude of the increase or decrease in AP firing caused by each modification, we calculated the mean and SE of the fold change in AP firing from the corresponding subsets of 244 and 1,068 simulations (Figure 6). Similar to the case with the nominal parameter set, of the four processes, the reduction of G_αq_-coupled receptor phosphorylation and G_αq_ activation rates yielded the highest increase and decrease in AP firing fold change, respectively, compared to the average fold change in the corresponding simulations without any modifications (Figure 6A, hatched bar representing *process 2* and *process 1* vs. open bar). Of the three proteins, Piezo2 knockout increased the AP firing fold change the most (Figure 6A, hatched bar representing Piezo2 vs. open bar), while TRPA1 knockout decreased the AP fold change (Figure 6A, hatched bar representing TRPA1 vs. open bar). However, the decrease was not as large as that observed in the simulation with the nominal parameter set. In addition, none of the modifications led to a statistically significant increase or decrease in the AP fold change compared to the nominal cases. The over-expression of either Piezo2, TRPA1, or Kv7.2 did not change the AP firing considerably. Overall, in the group of simulations representing protein over-expression and process up-regulation, none of the modifications considerably affected the AP firing fold change, except for a two-fold increase in the Nav1.7 and Nav1.8 phosphorylation rate that considerably decreased the AP firing fold change (Figure 6B, solid bar representing *process 4* vs. open bar). Overall, based on our simulation results, knocking out TRPA1 and reducing the rates of GPCR phosphorylation and G_αq_ activation had the largest effect on inflammation-induced changes in AP firing.

## Discussion

Inflammation present during musculoskeletal trauma induces transient hyperalgesia by sensitizing the nociceptive afferent neurons in the injured muscle tissue (Woolf and Ma, 2007). Unfortunately, in many cases, due to persistent inflammation, alterations in neuronal signaling, or both, these neurons remain in a sensitized state for a prolonged time, initiating the transition of acute pain to chronic pain. Due to the anatomical, biochemical, physiological, and functional heterogeneity among different neuron subpopulations, we still do not know the mechanisms or the specific alterations in membrane proteins that lead to or prolong an increased sensitization of muscle sensory neurons. Yet, identification of such key proteins/molecules and the specific alteration in their activities can facilitate the development of interventions that prevent pain chronification (Gold and Flake, 2005; Tsantoulas and McMahon, 2014; Waxman and Zamponi, 2014; Woolf, 2020). While there is evidence to suggest that inflammation induces alterations in the function and expression of some of the membrane proteins (e.g., TRPA1, Nav1.8, and Nav1.7) and intracellular signaling molecules (PKA and PKC) *in vitro* (Gold et al., 1998; Vijayaragavan et al., 2004; Gold and Flake, 2005), how these alterations affect pain signaling *in vivo* is difficult to predict, and the large number of plausible protein-signaling scenarios makes it impractical to identify such key membrane proteins that drive inflammation-induced sensitization through experimentation alone.

In this study, we used computational analysis to identify such key proteins and molecular processes. We first extended our validated model of a mouse muscle nociceptor to incorporate the effect of inflammation. The extended version accounts for the activity of 17 membrane proteins, four ER membrane proteins, and 28 second messenger molecules, including proteins, molecules, kinases, as well as Na^+^, K^+^, and Ca^2+^ ions, and describes 40 intracellular processes, including the activation and inactivation of the various membrane proteins as well as intracellular proteins and molecules by inflammatory mediators. We calibrated and validated the model using experimental data capturing the effects of inflammation mediators on neuronal sensitization (Aley and Levine, 1999; Hendrich et al., 2013; Khasabova et al., 2019). In agreement with experimental observations, in the presence of an inflammatory mediator, our model predicted a reduction in a nociceptor’s mechanical activation threshold and an increase in its AP firing rate due to mechanical forces (Figures 2B,C). To identify key regulators of neuronal sensitization, we used the model to simulate pain signaling responses to mechanical forces and an inflammatory mediator in 50,000 unique virtual muscle nociceptors, which were intended to represent the heterogeneity in protein expression and activity and the numerous plausible neuroplastic protein modifications that can occur *in vivo*. We found that modification of three ion channels (Kv7.2, Piezo2, and TRPA1) and four molecular processes (G_αq_-coupled receptor phosphorylation, PKA inhibition, G_αq_ activation, and both Nav1.8 and Nav1.7 phosphorylation) strongly regulated inflammation-induced increases in the total number of APs fired by the neurons. Moreover, by separately simulating the knockout or over-expression of each of the three proteins and by simulating an increase and decrease in the rates of each of the four processes, we showed that knocking out TRPA1 as well as reducing G_αq_-coupled receptor phosphorylation and G_αq_ activation rates had a greater effect on mechanically evoked AP firing during inflammation compared to other proteins and process modifications. Therefore, TRPA1 and G_αq_ subunit (specifically, enhanced G_αq_ activation) should be considered as potential targets for regulating inflammation-induced sensitization during musculoskeletal trauma.

### Key membrane proteins that regulate inflammation-induced sensitization

Neuronal sensitization is induced by a plethora of inflammatory mediators, including prostaglandins, bradykinins, cytokines, neurotrophins, serotonin, and histamine, which are released by both the afferent neurons themselves and the inflammatory cells present in injured tissues (Ciotu and Fischer, 2020). These mediators activate distinct signaling pathways within the neuron, although some of them share common pathways, e.g., both prostaglandins and bradykinin increase sensitization via activation of GPCRs (Gangadharan and Kuner, 2013). The majority of the distinct signaling pathways, however, converge within the neuron to increase the intracellular concentrations of proteins kinases, such as PKA, PKC, and mitogen-activated protein kinase, among others (Voscopoulos and Lema, 2010). The kinases ultimately evoke a change in the magnitude of neuronal AP firing by changing the expression or the currents through key Na^+^ and K^+^ ion channels, such as Nav1.8 and Nav1.7 (Vijayaragavan et al., 2004; Wu et al., 2012; Tanaka et al., 2016), Kv1.1 (Nicol et al., 1997; King et al., 2014; D’Adamo et al., 2020), and TRPA1 (Kwan et al., 2006; Karashima et al., 2009; del Camino et al., 2010), among others. Given that Nav1.8, Nav1.7, and Kv1.1 are modulated by many kinases, they have been investigated as targets to regulate pain, with limited success in certain specific cases (Kerstein et al., 2009; McDonnell et al., 2018; Ciotu and Fischer, 2020; D’Adamo et al., 2020; Woolf, 2020). Because a neuron’s AP firing rate is determined by a myriad of signaling processes, including membrane proteins and intracellular molecules, identifying specific regulatory proteins is challenging and requires understanding of their relative contributions to neuronal signaling. Thus, to address this challenge, we used the model to quantify the effect of each protein modification on inflammation-induced increase in the number of APs fired in thousands of distinct simulated neurons. Our analysis showed that in a majority of these simulated neurons, three proteins, TRPA1, Kv7.2, and Piezo2, strongly regulated the amount of increase in the total number of APs fired by the neuron after inflammation despite differential relative expression of activity of the other proteins and molecular processes in those neurons.

In addition, because we were interested in identifying proteins whose modifications could specifically regulate inflammation-induced increases in AP firing, even among the three key proteins, we singled out TRPA1 as the protein whose modifications could considerably reduce inflammation-induced sensitization, while not having a huge effect on the neuron’s response to mechanical stimuli in the absence of an inflammatory mediator. In Supplementary Table S4, we show, along with the fold change values, the number of APs fired in response to mechanical stimuli before and after the addition of an inflammatory mediator for the different modifications. Based on our results, the simulated knockout and over-expression of both Piezo2 (a recognized mechanosensitive channel) and Kv7.2 considerably changed the number of APs fired by the neuron (compared to the case with no modification) due to mechanical stimuli even before an inflammatory mediator was introduced. Therefore, these channels might be potential targets for regulating acute pain but may not specifically regulate the increase of inflammation-induced AP firing. However, in some cases, the addition of a Kv7.2 channel opener did in fact reduce inflammation-induced increases in the excitability of DRG neurons based on *in vitro* models of persistent peripheral neuropathic pain (Cisneros et al., 2015). In contrast to Piezo2 and Kv7.2 simulations, in the simulation of TRPA1 knockout, the AP firing before inflammation was close to the nominal case (12 APs vs. 14 APs), whereas the AP firing after the addition of an inflammatory mediator was considerably reduced (59 APs vs. 114 APs), suggesting that blocking or knocking out TRPA1 might help regulate inflammation-induced sensitization of these neurons. In agreement with our findings, TRPA1 blockers have been shown to reduce inflammatory pain initiated by afferent neurons in skin nerve preparations (Karashima et al., 2009; Kwan et al., 2009; del Camino et al., 2010).

### Key molecular processes that regulate inflammation-induced sensitization

In addition to membrane proteins, we also identified four processes downstream of inflammatory mediator-activated GPCR proteins that considerably affected the fold change of AP firing. Of the four processes, decreasing the G_αq_ activation rate caused the greatest increase in AP firing fold change compared to the simulation with no modifications (Figure 6A, hatched bar for *process 2*). We were surprised to observe that decreasing G_αq_ activation, which did indeed lead to a small decrease in PKC concentration, caused an increase in the number of APs fired in the model. However, when we examined the other model outputs to determine whether we could explain this result, we found that reducing G_αq_ activation in these simulations slowed the hydrolysis of membrane-bound phosphatidylinositol bisphosphate (PIP_2_), resulting in lower intracellular IP_3_ concentrations compared to the simulations with no modification (Putney and Tomita, 2012). The lower IP_3_ concentrations subsequently reduced the IP_3_-induced influx of Ca^2+^ into the intracellular compartment from the ER, which is one of the four ER mechanisms represented in the model that regulates intracellular Ca^2+^ concentration (Bennett et al., 2005). However, in response to this change, in the simulations with the G_αq_ modification, we observed that the Ca^2+^ fluxes by two of the other ER mechanisms (i.e., Ca^2+^-induced Ca^2+^ release via ryanodine receptors and Ca^2+^ leak via an ER leak channel) increased compared to simulations with no modification, while the Ca^2+^ uptake back into the ER via the sarcoendoplasmic reticulum calcium ATPase (the fourth mechanism) did not demonstrate a significant change (please refer to the Supplementary materials for the specific equations governing G_αq_ activation, PIP_2_ hydrolysis, and the intracellular IP_3_ and Ca^2+^ dynamics). The net effect of these altered ER mechanisms in the modified simulations was the higher intracellular Ca^2+^ concentration, which caused a higher depolarization of *V*_m_ in the neurons, ultimately leading to an increase in the number of fired APs. This is an example of the complexity and non-linearity of the intricate intracellular pathways, and how certain modifications will not always produce an expected change in the output. In contrast, for a different modification, we did see an expected outcome: decreasing the phosphorylation rate of G_αq_-coupled receptor caused the greatest decrease in the magnitude of fold change of AP firing (Figure 6A, hatched bar for *process 1*). GPCRs are the largest group of sensory receptors present on nociceptors and play an important role in inflammatory nociception, making them an attractive target for interventions aimed at reducing inflammatory pain (Sun and Ye, 2012). In fact, mice lacking a specific type of G_αi_ receptor subunit have been reported to display altered pain perception and inflammatory responses (Doi et al., 2002). However, because GPCRs have overlapping functions in many other tissues, illustrated by the fact that one-third of all U.S. Food and Drug Administration-approved drugs target GPCRs (Retamal et al., 2019), exploring their role as an analgesic drug has been challenging. Other studies have shown that targeting the downstream effectors of GPCR activation, such as adding PKC and PKA inhibitors to the neurons, could reduce the effects of PGE_2_-induced sensitization in DRG neurons *in vitro* (Gold et al., 1998; Gold and Gebhart, 2010). Indeed, even in our computational analysis, we showed that increasing the inactivation rates of both PKC and PKA are key for regulating inflammation-induced increases in the total number of APs fired by a neuron (Figure 4; Supplementary Figure S1).

While previous studies have highlighted the role of individual ion channels or protein kinases in regulating inflammatory pain, by using computational analysis we were able to evaluate the relative effect of modifying many different proteins and kinases, one at a time, on AP firing in the same set of 50,000 simulated neurons, which is not feasible in experimental studies. For example, in a group of 244 neurons where the average inflammation-induced AP firing fold change with no modification was 6 (±1), when we modified them one at a time by changing the corresponding parameter values in the model, of seven modifications only two strongly altered the average AP fold change after inflammation (Figure 6A, hatched bars for *Processes 1* and *2*). Specifically, a 10-fold reduction of GPCR phosphorylation rate decreased the average AP fold change to 2 (±1), while a 10-fold reduction of the G_αq_ activation rate increased it to 12 (±4). Thus, in addition to identifying a panel of key proteins and molecules that could be potential targets for regulating the level of neuronal excitability induced by inflammatory mediators, our analysis also provided a one-to-one comparison of the efficacy of targeting each key protein or process in the same population of neurons.

### Assumptions and limitations

Our computational model has several limitations arising from simplifying assumptions required to capture the complex nature of inflammation-induced sensitization in muscle afferent neurons. First, due to limited availability of electrophysiological data for inflammation-induced sensitization in mice, we used data from rat neurons to calibrate the model, which might affect model accuracy. However, we did validate the model by comparing its predictions with data from mouse neurons. Second, we only modeled two specific GPCR signaling pathways that are activated by a subset of the inflammatory mediators that can be present in an injured tissue. It is possible that proteins and molecules involved in pathways which we do not currently account for might be important for regulating inflammation-induced sensitization. However, as previously discussed, because many inflammatory pathways are known to converge within the neuron, resulting in an increase in the concentrations of PKC and PKA (Gold and Flake, 2005; Gold and Gebhart, 2010; Gangadharan and Kuner, 2013; Pak et al., 2018) whose effects we do currently model, we could incorporate the effect of other inflammatory mediators if and when relevant. Moreover, while active PKC and PKA are present in basal concentrations within the neuron, in the model we set their initial concentrations to zero because we assumed that basal levels of PKA and PKC do not considerably affect inflammation-induced sensitization.

Third, in our model we adopted many parameter values from previous computational studies developed to describe neurons from animals other than mice or from physiological tissues other than muscle (Bennett et al., 2005; Lindskog et al., 2006; Mandge and Manchanda, 2018). While we performed a validation procedure to match our computational simulations to experimental data recorded from mouse neurons, we did not directly derive parameters from single ion channel current measurements in mouse neurons. This simplification could impact the accuracy of certain model parameters. Fourth, while we have incorporated the description of the relevant channels and intracellular molecules involved in the transduction of inflammation-induced sensitization in muscle nociceptors, our model does not represent all possible channels and their isomers that are present on the neuronal membrane, or every enzyme or kinase present within the neuron (Woolf and Ma, 2007; Dubin and Patapoutian, 2010; Mense, 2010). Therefore, there is a possibility that a channel or a specific isomer of a channel currently not included in the model could still be a key regulator of inflammatory pain in muscles. Finally, our hypotheses regarding the contributions of TRPA1, Kv7.2, Piezo2, GPCR phosphorylation, G_αq_ activation, PKA inactivation, and both Nav1.8 and Nav1.7 phosphorylation to the sensitization of muscle nociceptors stem solely from simulations. These hypotheses need to be tested by independent mice experiments, where we separately modify each protein or process in the presence of an inflammatory mediator and assess the effect of the modification on AP firing. Ultimately, there is always the question of translatability of nociceptive mechanisms across species. Until we can reliably perform *in vivo* investigations on human nociceptors, mouse models provide the opportunity to use genetic approaches to investigate the molecular mechanisms of nociceptive signaling. Importantly, rodents and humans are known to exhibit certain similarities in terms of nociceptive responses, such as the functional organization of the spinal cord, the ability of sensory neurons to alter their thresholds, and the ability to sensitize following repetitive injuries (Fitzgerald, 2005; Toossi et al., 2021).

## Conclusion

The identification of transmembrane proteins and other molecules that regulate inflammation-induced sensitization of peripheral nociceptive afferent neurons is challenging, stemming from the heterogeneity in afferent neuron types and functions across different tissues and species and the plethora of inflammatory mediators that can act upon the neurons. In this study, we specifically focused on the effect of inflammation on mechanical nociception in muscle tissue. To this end, we developed a computational model of a muscle mechanosensitive nociceptor in which we incorporated two inflammation-activated signaling pathways that heightened its AP response to mechanical forces. Our results allowed us to hypothesize that: (1) TRPA1, Kv7.2, and Piezo2 as well as G_αq_ activation and PKA inactivation are regulators of inflammation-induced increases in the magnitude of AP firing by muscle nociceptors; (2) increasing the G_αq_-coupled receptor phosphorylation rate and decreasing the G_αq_ activation rate further increase inflammation-induced AP firing; and (3) TRPA1 knockout decreases the magnitude of AP firing.

Our findings could be used to advance the field in different ways. First, *in vivo* studies could be performed to experimentally test our computationally derived hypotheses, which, if confirmed, would lead to improved understanding of acute pain initiation and inflammation-induced sensitization in muscle tissue. Second, animal experiments to assess behavioral responses could help us understand how neuronal inhibition or activation of the identified proteins in living animals correlate with pain behaviors (e.g., paw withdrawal in response to force). Third, we could use our findings to assess whether the same proteins that sensitize muscle neurons are also involved in different pathological pain scenarios, such as pain arising from direct nerve injury (i.e., neuropathic pain). Finally, we could extend our computational model to include the kinetics of pharmaceutical drugs that act as inhibitors or enhancers for specific ion channels or receptors and predict their efficacy in reducing neuronal sensitization in a dose-dependent manner.

## Data availability statement

The original contributions presented in the study are included in the article/Supplementary material, further inquiries can be directed to the corresponding author.

## Author contributions

SN, SGT, and JR conceptualized the work. SN developed the model and performed the computational analysis. SGT assisted in the computational analysis. SN and JR wrote the manuscript. All authors contributed to the article and approved the submitted version.

## Funding

This work was supported by the U.S. Army Medical Research and Development Command under Contract no. W81XWH20C0031.

## Conflict of interest

SN and SGT were employed by The Henry M. Jackson Foundation for the Advancement of Military Medicine, Inc.

The remaining author declares that the research was conducted in the absence of any commercial or financial relationships that could be construed as a potential conflict of interest.

## Publisher’s note

All claims expressed in this article are solely those of the authors and do not necessarily represent those of their affiliated organizations, or those of the publisher, the editors and the reviewers. Any product that may be evaluated in this article, or claim that may be made by its manufacturer, is not guaranteed or endorsed by the publisher.

## Author Disclaimer

The opinions and assertions contained herein are the private views of the authors and are not to be construed as official or as reflecting the views of the United States (U.S.) Army, the U.S. Department of Defense, or The Henry M. Jackson Foundation (HJF) for the Advancement of Military Medicine, Inc. This paper has been approved for public release with unlimited distribution.
