# Supplementary material for: Identification of key factors driving inflammation-induced sensitization of muscle sensory neurons
Source: Front Neurosci. 2023 May 12;17:1147437. doi: 10.3389/fnins.2023.1147437 (PMC10213456; doi:10.3389/fnins.2023.1147437)
Supplement: Supplementary file 1 [file Data_Sheet_1.pdf]

SUPPLEMENTARY MATERIAL

Identification of key factors driving inflammation-induced sensitization of muscle sensory neurons

by Sridevi Nagaraja, Shivendra G. Tewari, and Jaques Reifman

Inflammation-activated G protein-coupled receptor pathways described in the model

Protein kinase C pathway

In this mechanism, we described the kinetics of $G_{\alpha q}$, which when activated by an inflammatory mediator dissociates into two subunits. One of the active subunits, G_{α}, recruits inactive phospholipase (PLC) enzyme from the cytosol to form active PLC at the membrane. PLC then hydrolyzes membrane-bound phosphatidylinositol 4,5-bisphosphate (PIP_2), leading to the production of two intracellular second messengers, inositol trisphosphate (IP_3) and diacylglycerol (DAG). DAG activates protein kinase C (PKC) in the cytosol, and the active PKC ultimately modifies the function of ion channels on the neuronal membrane (Gold and Flake, 2005). In our model, we described the kinetics of the receptor, enzymes, and second messenger molecules using equations and parameters described previously (Bennett et al., 2005; Kapela et al., 2008; Mohan et al., 2017).

Protein kinase A pathway

In this mechanism, we described the kinetics of $G_{\alpha s}$, which when activated by an inflammatory mediator dissociates into three subunits. The G_{α} subunit recruits adjacent membrane-bound adenylate cyclase (AC), which then catalyzes the conversion of ATP in the cytosol to cAMP, which acts as a second messenger and activates protein kinase A (PKA), among other enzymes (Li et al., 2019). In our model, we described the kinetics of each of these receptor subunits, enzymes, and second messenger molecules, using equations and parameters described previously (Lindskog et al., 2006; Leander and Friedman, 2014).

Table S1. Model variable name, description, and initial value

Variable name	Description	Initial value	Variable name	Description	Initial value
$Nav1.8_m$	Activation constant of voltage-gated Nav1.8 channel	0	$TREK_m$	Activation constant of two-pore K^+ channel	0
$Nav1.8_h$	Inactivation constant of voltage-gated Nav1.8 channel	1	Ka_n	Activation constant of M-type K^+ channel	0
$Nav1.7_m$	Activation constant of voltage-gated Nav1.7 channel	0	Ka_{hfast}	Fast inactivation constant of M-type K^+ channel	1
$Nav1.7_h$	Inactivation constant of voltage-gated Nav1.7 channel	1	Ka_{hslow}	Slow inactivation constant of M-type K^+ channel	1
$Nav1.9_m$	Activation constant of voltage-gated Nav1.9 channel	0	$Kv7_n$	Activation constant of voltage-gated Kv7.2 channel	0
$Nav1.9_h$	Inactivation constant of voltage-gated Nav1.9 channel	1	KDR_n	Activation constant of delayed rectifying K^+ channel	0
$Piezo_m$	Fast activation constant of Piezo2 channel	0	$BKCa_n$	Activation constant of large-conductance Ca^{2+}-activated K^+ channel	0
$Piezo_h$	Inactivation variable of Piezo2 channel	1	CaL_m	Activation constant of L-type voltage-gated Ca^{2+} channel	0
$ASIC3_m$	Activation constant of ASIC3 channel	0	CaL_h	Inactivation constant of L-type voltage-gated Ca^{2+} channel	1
$ASIC3_h$	Inactivation constant of ASIC3 channel	1	CaT_m	Activation constant of T-type voltage-gated Ca^{2+} channel	0
$TRPA1_m$	Activation constant of TRPA1 channel	0	CaT_h	Inactivation constant of T-type voltage-gated Ca^{2+} channel	1
$TRPA1_h$	Inactivation constant of TRPA1 channel	1	V_m	Membrane potential	-55 mV
$[Na_i^+]$	Intracellular Na^+ concentration	14 mM	$[K_i^+]$	Intracellular K^+ concentration	140 mM
$[Ca_i^{2+}]$	Intracellular free calcium ion concentration	5×10^{-5} mM	$[Ca_{ER}^{2+}]$	Calcium concentration in the endoplasmic reticulum	0.25 mM
IP_3	Intracellular inositol trisphosphate concentration	1×10^{-5} mM	hIP_3	Activation constant of the IP_3 receptor	0.667

$G_{\alpha qunphos}$	Total number of unphosphorylated $G_{\alpha q}$ receptors	1.7×10^{5}	$[G_{\alpha sact}]$	Concentration of α subunits of $G_{\alpha s}$ receptors	0
$G_{\alpha qphos}$	Total phosphorylated $G_{\alpha q}$ receptors	0	$[G_{\alpha s\beta\gamma}]$	Concentration of αβ subunits of $G_{\alpha s}$ receptors	0
$G_{\alpha q}$	Number of α subunits of $G_{\alpha q}$ receptors	0	$[G_{\beta\gamma s}]$	Concentration of β subunits of $G_{\alpha s}$ receptors	0
[DAG]	Intracellular diacylglycerol concentration	0 mM	[cAMP]	Intracellular concentrations of cyclic adenosine monophosphate	0 mM
PIP_2	Total number of phosphatidylinositol 4,5-bisphosphate	5×10^{7}	[RC]	Intracellular concentration of inactive PKA	2.2×10^{-4} mM
PLC	Concentration of active phospholipase C	0 mM	$[R_{cAMP}]$	Intracellular concentration of PKA regulatory subunit	0 mM
$[DAG_{PKC}]$	Intracellular concentration of DAG-PKC complex	0 mM	$[RC_{caMP2}]$	Intracellular concentration of intermediate regulatory PKA catalytic subunit	0 mM
PLC_{inact}	Concentration of membrane-bound inactive phospholipase C	1×10^{-2} mM	$[RC_{cAMP4}]$	Intracellular concentration of intermediate regulatory PKA-PKA catalytic subunit	0 mM
$[PKC_{inact}]$	Intracellular concentration of inactive protein kinase C	1×10^{-3} mM	[PKA]	Intracellular concentration of active protein kinase A	0 mM
[PKC]	Intracellular concentration of protein kinase C	0 mM	$V_{actNav1.7}$	Membrane potential threshold for Nav1.7 activation	-25.8 mV
$V_{actNav1.8}$	Membrane potential threshold for Nav1.8 activation	-11.4 mV	$V_{inactNav1.7}$	Membrane potential threshold for Nav1.7 inactivation	55.8 mV
$V_{inactNav1.8}$	Membrane potential threshold for Nav1.8 inactivation	-24.6 mV	$M_{actTRPA1}$	Mechanical force threshold for TRPA1 activation	40 mN
$V_{actKv1.1}$	Membrane potential threshold for Kv1.1 activation	-35 mV			

Table S2. Model parameter number (used in the model), name, description, value, units, and sources

P#	Parameter name	Description	Value	Unit	References
**Na^+-Ca^{2+}exchanger**					
1	kNCX	Constant for NCX	25.85		(Nagaraja et al., 2021)
2	kNa	Half-saturation constant for extracellular [Na^+]	87.50	mM	
3	kCa	Half-saturation constant for extracellular [Ca^{2+}]	1.38	mM	
4	$Imax_{NCX}$	Maximum current density	1×10^5	nS	
**ASIC3 channel**					
5	Vh_{ASIC}	Half-activation pH for activation factor	6.202	pH	(Nagaraja et al., 2021)
6	$k_{act}ASIC$	Steepness factor of activation	0.1754	pH	
7	τact_{ASIC}	Hill slope of activation factor	5.000	ms	
8	Vs_{ASIC}	Half-inactivation pH for activation factor	7.061	pH	
9	$k_{inact}ASIC$	Steepness factor of inactivation	0.0452	pH	
10	$Imax_{ASIC1\beta}$	Maximum conductance of ASIC channel	15.0	nS	
**Na^+-K^+ pump**					
11	nH_{Na}	Hill coefficient for sodium and potassium	1.5		(Nagaraja et al., 2021)
12	KNa_{NaK}	Binding constant for intracellular [Na^+]	14.5	mM	
13	KK_{NaK}	Binding constant for extracellular [K^+]	1.5	mM	
14	$Imax_{NaK}$	Maximum current density	150	pA	
**Piezo2 channel**					
15	Vh_{Piezo}	Half-activation force for inactivating factor	0.9	mN	(Nagaraja et al., 2021)
16	Vs_{Piezo}	Half-activation force for inactivating factor	0.6	mN	
17	$k1_{inact}Piezo$	Steepness factor of inactivation	0.3	mN	
18	$k2_{inact}Piezo$	Steepness factor of inactivation	0.1	mN	
19	τact_{Piezo}	Time constant for activation factor	1	ms	
20	$\tau 1inact_{Piezo}$	Time constant for fast inactivation factor	3	ms	
21	$Imax_{Piezo}$	Maximum conductance of Piezo channel	40	nS	
**TREK-1 channel**					
22	Vm_{TREK}	Half-activation force for activation factor	8	mN	(Nagaraja et al., 2021)
23	$kact_{TREK}$	Steepness factor of activation	1	mN	
24	τact_{TREK}	Activation time constant	1	ms	
25	$Imax_{TREK}$	Maximum TREK channel conductance	0.5	nS	
**TRPA1 channel**					
26	$kact_{TRPA1}$	Steepness factor of activation	20	mN	(Nagaraja et al., 2021)
27	Vh_{TRPA1}	Half-activation force for inactivation factor	40	mN	
28	$kinact_{TRPA1}$	Steepness factor of inactivation	20	mN	
29	τact_{TRPA1}	Activation time constant	1	ms	
30	$\tau inact_{TRPA1}$	Inactivation time constant	5	ms	
31	$Imax_{TRPA1}$	Maximum conductance	15	nS	
**Kv7.2**					
32	$Imax_{Kv7}$	Maximum Kv7 conductance	600	nS	Modified
33	$k1act_{Kv7}$	Steepness factor of activation	0.00395	mV	(Nagaraja et al., 2021)
34	Vm_{Kv7}	Half-activation membrane potential for activation factor	15	mV	
35	$k2act_{Kv7}$	Steepness factor of activation	40	mV	
36	$k1inact_{Kv7}$	Steepness factor of inactivation	0.00395	mV	
37	$k2inact_{Kv7}$	Steepness factor of inactivation	20	mV	

38	τact_{Kv7}	Activation time constant	3	ms	Modified
**KDR channel**					
39	$Imax_{KDR}$	Maximum KDR conductance	200	nS	(Nagaraja et al., 2021)
40	δ_{KDR}	Activation factor	0.577	mV	
41	$kact_{KDR}$	Steepness factor of activation	15.4	mV	
42	τact_{TRPA1}	Activation time constant	300	ms	Modified
**A-type K^+ channel**					
43	$Imax_{Ka}$	Maximum A-type K^+ channel conductance	6	nS	Modified
**Nav1.8 channel**					
44	$Imax_{Nav1.8}$	Maximum Nav1.8 channel conductance	150	nS	Modified
45	$kact_{Nav1.8}$	Steepness factor of activation	8.75	mV	(Nagaraja et al., 2021)
46	$kinact_{Nav1.8}$	Steepness factor of inactivation	5.76	mV	
**Nav1.9 channel**					
47	$Imax_{Nav1.9}$	Maximum Nav1.9 channel conductance	0.5	nS	Modified
**Nav1.7 channel**					
48	$Imax_{Nav1.7}$	Maximum Nav1.7 channel conductance	212.0	nS	(Nagaraja et al., 2021)
49	$kact_{Nav1.7}$	Steepness factor of activation	7.8	mV	
50	$kinact_{Nav1.7}$	Steepness factor of inactivation	8.9	mV	
**Potassium leak channel**					
51	$Imax_{Kleak}$	Leak channel conductance	0.6	nS	Modified
**Ca^{2+}-activated K^+ channel**					
52	$Imax_{BKCa}$	Maximum BKCa channel conductance	10	nS	(Nagaraja et al., 2021)
**L-type voltage-gated Ca^{2+} channel (VGCC)**					
53	$Imax_{LCa}$	Maximum L-type VGCC conductance	10	nS	(Nagaraja et al., 2021)
54	Vm_{LCa}	Half-activation potential for activation factor	-22.8	mV	
55	$kact_{LCa}$	Steepness factor for activation	9.85	mV	
56	Vh_{LCa}	Half-activation potential for inactivation factor	-34.61	mV	
57	$kinact_{LCa}$	Steepness factor for inactivation	5.95	mV	
58	τact_{LCa}	Time constant for activation	2.38	ms	
59	$\tau inact_{LCa}$	Time constant for inactivation	25.2	ms	
**T-type voltage-gated Ca^{2+} channel**					
60	Vm_{TCa}	Half-activation potential for activation factor	-25.0	mV	(Nagaraja et al., 2021)
61	$Kact_{TCa}$	Steepness factor for activation	-5.0	mV	
62	Vh_{TCa}	Half-activation potential for inactivation factor	-38.0	mV	
63	$Kinact_{TCa}$	Steepness factor for inactivation	-5.0	mV	
64	τact_{TCa}	Time constant for activation	1	ms	
65	$\tau inact_{TCa}$	Time constant for inactivation	409	ms	
66	$Imax_{TCa}$	Maximum T-type VGCC conductance	0.099	nS	
**IP_3 receptor (IP_3R)**					
67	$Imax_{IP3R}$	Rate constant of Ca^{2+} release by IP_3R	0.00288	nS	(Nagaraja et al., 2021)
68	$kdis_{IP3}$	Dissociation constant for IP_3 binding to IP_3R	2.7	mM	
69	$kdisinact_{Ca}$	Dissociation constant for Ca^{2+} inactivation	1.0×10^{-4}	mM	
70	$kdisact_{Ca}$	Dissociation constant for Ca^{2+} activation	1.7×10^{-4}	mM	
71	k_{Ca}	Rate of Ca^{2+} binding to the inhibitory site	0.0003	mM s^{-1}	
**PMCA pump**					
72	$Imax_{PMCA}$	Maximum current	7.6418	pA	(Nagaraja et al., 2021)
73	KCa_{PMCA}	Michaelis constant	0.1562	mM	
**SERCA pump**					

74	KCa_{SERCA}	Michaelis constant	3.94×10^{-4}	mN	(Nagaraja et al., 2021)
75	$Imax_{SERCA}$	Maximum SERCA uptake	0.002	pA	
**Ryanodine receptor (RyR)**					
76	KCa_{CICR}	Minimum intracellular [Ca^{2+}] for RyR activation	1.2×10^{-4}	mM	(Nagaraja et al., 2021)
77	$Imax_{CICR}$	Rate constant of Ca^{2+} release by RyR	5.03×10^{-4}	pA	
78	kd_{CICRCa}	Dissociation constant for Ca^{2+} inactivation	0.0501	mM	
**Endoplasmic reticulum (ER) Ca^{2+} leak**					
79	$Imax_{ERleak}$	Maximum passive leak from ER	3.03×10^{-5}	pA	(Nagaraja et al., 2021)
**Calcium buffering in ER**					
80	K_{CQSN}	Binding affinity of calsequestrin	1.21	mM	(Nagaraja et al., 2021)
81	CQSN	Concentration of calsequestrin in ER	16.0	mM	
**Protein kinase C (PKC) activation and signaling**					
82	RTG	Total unphosphorylated $G_{\alpha q}$ receptors	2.00×10^{4}		(Bennett et al., 2005; Mohan et al., 2017)
83	K_{1G}	Unphosphorylated receptor dissociation constant	0.01	mM	
84	K_{2G}	Phosphorylated receptor dissociation constant	0.2	mM	
85	k_{rG}	Receptor recycling rate	1.75×10^{-7}	ms^{-1}	
86	k_{pG}	Receptor phosphorylation rate	1.00×10^{-3}	ms^{-1}	
87	k_{eG}	Receptor endocytosis rate	6.00×10^{-6}	ms^{-1}	
88	$epsilon_G$	Fraction of mobile receptors	0.85		
89	GTG	Total activated $G_{\alpha q}$ receptors	1.00×10^{5}		
90	k_{degG}	IP_3 degradation rate	0.00125	ms^{-1}	
91	k_{aG}	$G_{\alpha q}$ subunit activation rate	1.70×10^{-4}	ms^{-1}	
92	k_{dG}	$G_{\alpha q}$ subunit deactivation rate	1.50×10^{-3}	ms^{-1}	
93	$PIP2_T$	Total PIP_2 molecules	5.00×10^{7}		
94	r_{rG}	PIP_2 replenishment rate	1.50×10^{-5}	ms^{-1}	
95	k_{cG}	Dissociation constant for Ca^{2+} binding to PLC	4.00×10^{-4}	mM	
96	α_G	Effective signal gain parameter	2.78×10^{-8}	ms^{-1}	
97	γ_G	Coefficient to convert number of molecules to concentration	6.00×10^{8}		
98	k_{PLCact}	PLC activation rate	2.2967	$mM^{-1}\cdot ms^{-1}$	Modified
99	$k_{PLCinact}$	PLC inactivation rate	0.3389	ms^{-1}	
100	k_{hyd}	Rate of diacylglycerol (DAG) activation by PIP_2	4.99×10^{-10}	$mM^{-2}\cdot ms^{-1}$	(Mohan et al., 2017)
101	k_{deg}	DAG degradation rate	0.0499	ms^{-1}	
102	k_{actPKC}	Rate of PKC activation by DAG	0.20	$mM^{-1}\cdot ms^{-1}$	Modified
103	$k_{inactPKC}$	PKC inactivation rate	0.0022	ms^{-1}	
104	k_{off}	Rate of dissociation of DAG-PKC complex	8.00×10^{-3}	ms^{-1}	
105	k_{dp}	Rate of association of DAG and PKC	0.0112	ms^{-1}	
**Protein kinase A (PKA) activation and signaling**					
106	AC_{tot}	Total basal concentration of adenyl cyclase	2.90×10^{-5}	mM	(Leander and Friedman, 2014)
107	$GPCR_{tot}$	Basal concentration of total phosphorylated $G_{\alpha s}$ receptors	9.70×10^{-6}	mM	
108	$G_{\alpha stot}$	Total activated of $G_{\alpha s}$ subunits	0.0061		
109	k_{EPdiss}	Dissociation rate constant	1.90×10^{-5}		
110	$k1_{G\alpha s}$	$G_{\alpha s}$ activation rate	5.00×10^{-3}	ms^{-1}	
111	$k2_{G\alpha s}$	$G_{\alpha s}$ hydrolysis rate	7.00×10^{-5}	ms^{-1}	
112	$k3_{G\alpha s}$	$G_{\alpha s}$-$\beta\gamma$ association rate	0.7	$mM^{-1}\cdot ms^{-1}$	
113	$k4_{G\alpha s}$	$G_{\alpha s}$-$\beta\gamma$ disassociation rate	1.89×10^{-5}	ms^{-1}	
114	k_{ACdiss}	Dissociation rate constant	3.60×10^{-6}	mM	

115	$kAC_{\beta\gamma diss}$	Disassociation constant for AC and βγ	9.00×10^{-5}	mM	
116	$k5_{G\alpha s}$	Active cAMP production rate	0.0105	ms^{-1}	
117	$k6_{G\alpha s}$	Basal cAMP production rate	3.5×10^{-4}	ms^{-1}	
118	$k_{degcAMP}$	cAMP degradation rate	0.013	ms^{-1}	
119	K_{fPKA}	Rate of association of PKA and cAMP	1.30×10^{-2}	$mM^{-2}\cdot ms^{-1}$	(Lindskog et al., 2006)
120	K_{bPKA}	Rate of disassociation of RCcaMP2	6.00×10^{-6}	ms^{-1}	
121	kf_9	Rate of association of cAMP and RCcaMP2	1.73×10^{-2}	$mM^{-2}\cdot ms^{-1}$	
122	kb_9	Rate of disassociation of RCcaMP4	6.00×10^{-5}	ms^{-1}	
123	kf_{10}	Rate of disassociation of RC and RCcaMP	8.00×10^{-6}	ms^{-1}	
124	kb_{10}	Rate of association of RC and RCcaMP	4.8	$mM^{-2}\cdot ms^{-1}$	
**TRPA1 phosphorylation by PKA and PKC**					
125	$k_{halfPKC}$	Phosphorylation factor of PKC	5.47×10^{-6}	mV	Modified
126	$k_{slopePKC}$	Steepness factor of PKC	1.84×10^{-5}	mV	
127	$k_{halfPKA}$	Phosphorylation factor of PKA	3.47×10^{-5}	mV	
128	$k_{slopePKA}$	Steepness factor of PKA	8.39×10^{-6}	mV	
129	$\tau_{TRPA1phos}$	TRPA1 phosphorylation time constant	5×10^{6}	ms^{-1}	
130	$\tau_{Kv1.1phos}$	Kv1.1 phosphorylation time constant	5×10^{6}	ms^{-1}	
131	$\tau_{Navphos}$	Nav1.8 and Nav1.7 phosphorylation time constant	5×10^{6}	ms^{-1}	

Figure S1

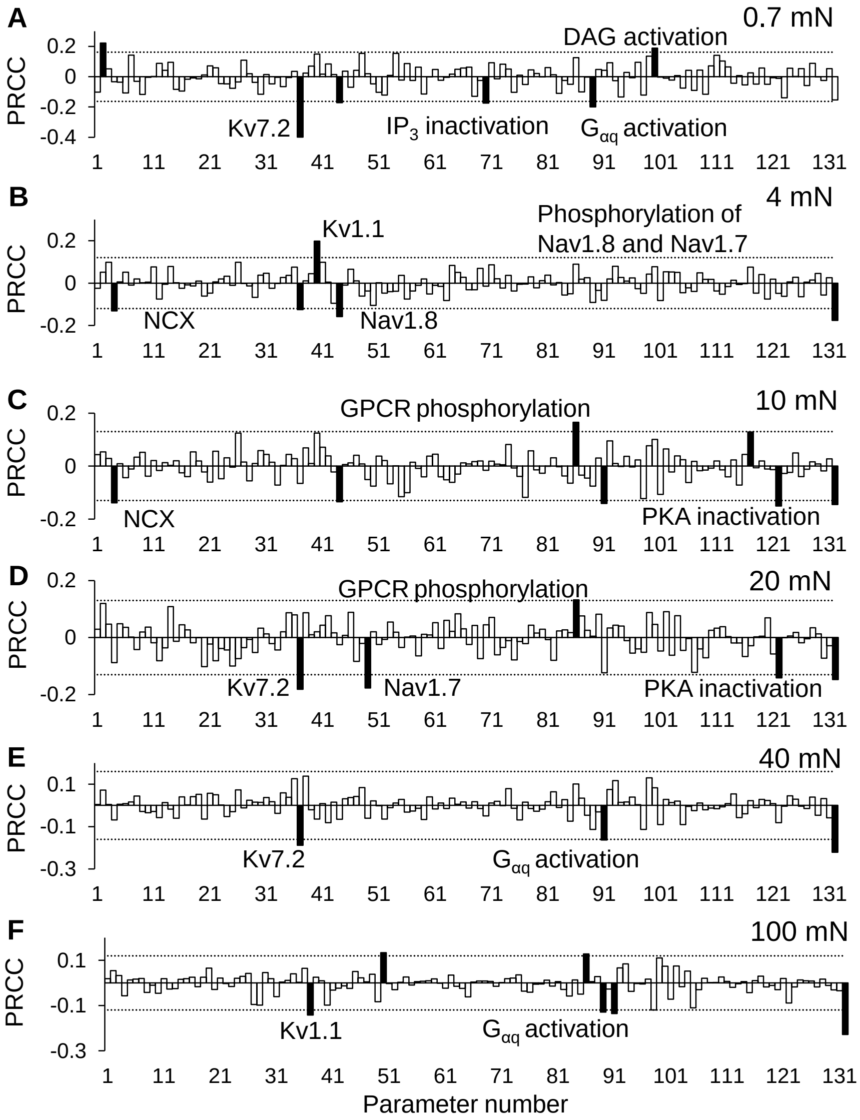

Figure S1. Partial rank correlation coefficient (PRCC) analysis identified key proteins and processes for action potential (AP) regulation. The bars show the PRCC values between the 131 model parameters and the fold changes in the number of APs fired after the separate application of a series of six mechanical forces of (A) 0.7 mN, (B) 4 mN, (C) 10 mN, (D) 20 mN, (E) 40 mN, and (F) 100 mN computed from simulations in which inflammation increased AP firing. The PRCCs above their respective thresholds (dotted horizontal lines) that were statistically significant (i.e., $p < 0.01$) are indicated by solid black bars, and the labels of the bars show the ion channels/ion pumps or the rates of intracellular processes that these parameters describe in the model. The number of simulations in which the number of APs fired increased after inflammation were 306, 837, 1,007, 529, 1,319, and 2,575 for the applied mechanical forces of 0.7, 4, 10, 20, 40, and 100 mN, respectively. DAG, diacylglycerol; GPCR, G protein-coupled receptor; IP_3, inositol trisphospate.

Figure S2

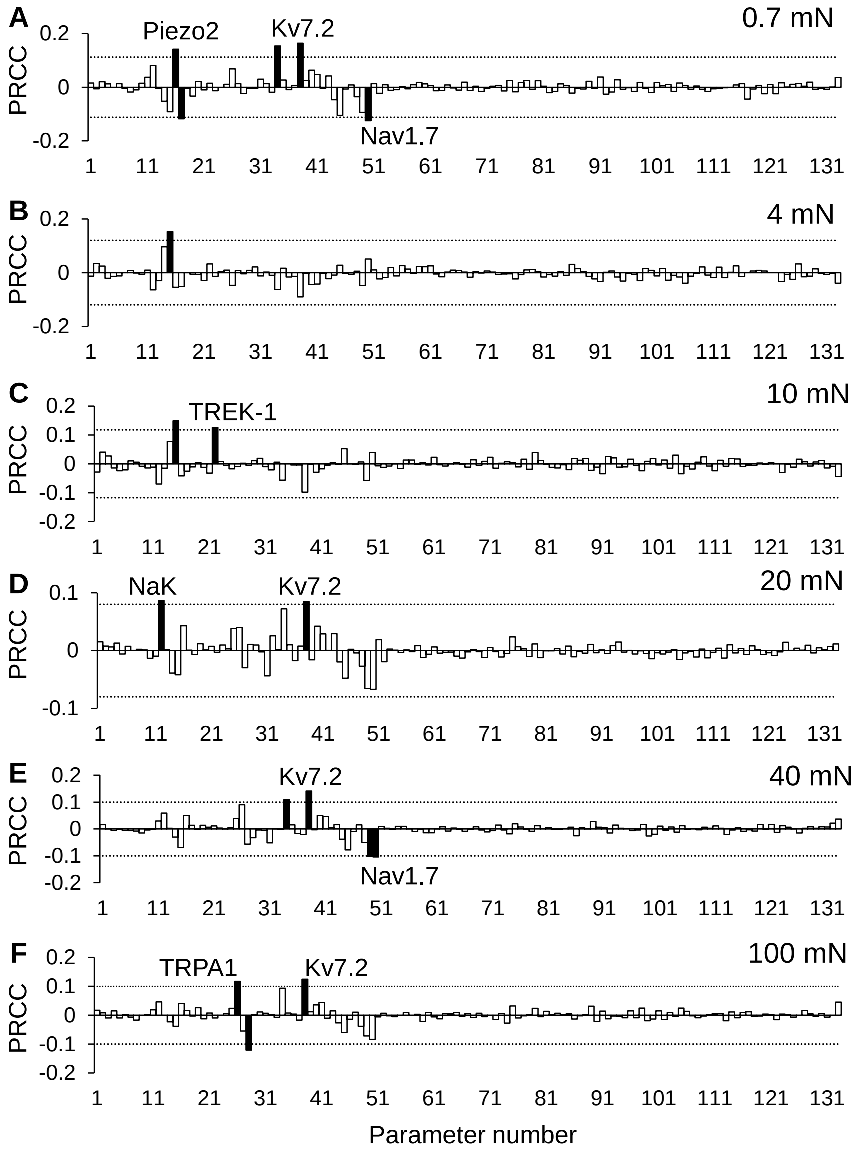

Figure S2. Partial rank correlation coefficient (PRCC) analysis identified key proteins and processes for action potential (AP) regulation. The bars show the PRCC values between the 131 model parameters and the fold changes in the number of APs fired after the separate application of a series of six mechanical forces of (A) 0.7 mN, (B) 4 mN, (C) 10 mN, (D) 20 mN, (E) 40 mN, and (F) 100 mN computed from simulations in which inflammation decreased AP firing. The PRCCs above their respective thresholds (dotted horizontal lines) that were statistically significant (i.e., $p < 0.01$) are indicated by solid black bars, and the labels of the bars show the ion channels/ion pumps or the rates of intracellular processes that these parameters describe in the model. The number of simulations in which the number of APs fired decreased after inflammation were 6,134, 13,625, 13,626, 14,484, 14,311, and 17,509 for the applied mechanical forces of 0.7, 4, 10, 20, 40, and 100 mN, respectively.

Table S3. List of Bhattacharyya coefficients for the model's 131 parameters, computed using the parameter distributions in the simulations of sensitized and non-sensitized neurons

Parameter number	Bhattacharyya coefficient	Parameter number	Bhattacharyya coefficient	Parameter number	Bhattacharyya coefficient
1	0.994076	45	0.986364	89	0.988439
2	0.992218	46	0.993500	90	0.992524
3	0.992158	47	0.993177	91	0.986838
4	0.993218	48	0.990526	92	0.990516
5	0.993584	49	0.948802	93	0.989588
6	0.993606	50	0.972886	94	0.991931
7	0.993911	51	0.992912	95	0.992648
8	0.994278	52	0.992366	96	0.992518
9	0.991266	53	0.989942	97	0.991408
10	0.993573	54	0.993005	98	0.988138
11	0.992801	55	0.993110	99	0.988453
12	0.985838	56	0.993234	100	0.990976
13	0.993620	57	0.993576	101	0.990088
14	0.986760	58	0.994211	102	0.989887
15	0.965129	59	0.993072	103	0.992670
16	0.992167	60	0.992288	104	0.990117
17	0.990052	61	0.994663	105	0.991441
18	0.990342	62	0.991923	106	0.993061
19	0.991623	63	0.994803	107	0.991699
20	0.993535	64	0.993847	108	0.992943
21	0.992141	65	0.992111	109	0.992606
22	0.992966	66	0.992985	110	0.994406
23	0.993625	67	0.993741	111	0.993511
24	0.992183	68	0.993265	112	0.991349
25	0.992039	69	0.992306	113	0.993604
26	0.984754	70	0.992138	114	0.993472
27	0.992974	71	0.992832	115	0.992237
28	0.992090	72	0.992988	116	0.992598
29	0.992472	73	0.994244	117	0.994399
30	0.992483	74	0.993390	118	0.993215
31	0.991196	75	0.990438	119	0.993589
32	0.991266	76	0.993124	120	0.993410
33	0.993417	77	0.992430	121	0.992297
34	0.976274	78	0.992000	122	0.993004
35	0.993259	79	0.991922	123	0.989626
36	0.994324	80	0.990773	124	0.992886
37	0.990319	81	0.993283	125	0.992653
38	0.971447	82	0.993563	126	0.993665
39	0.991562	83	0.991318	127	0.993665
40	0.990489	84	0.991416	128	0.993471
41	0.992677	85	0.991646	129	0.992239
42	0.993160	86	0.984570	130	0.991636
43	0.993396	87	0.994010	131	0.977239
44	0.991408	88	0.992848		

Figure S3

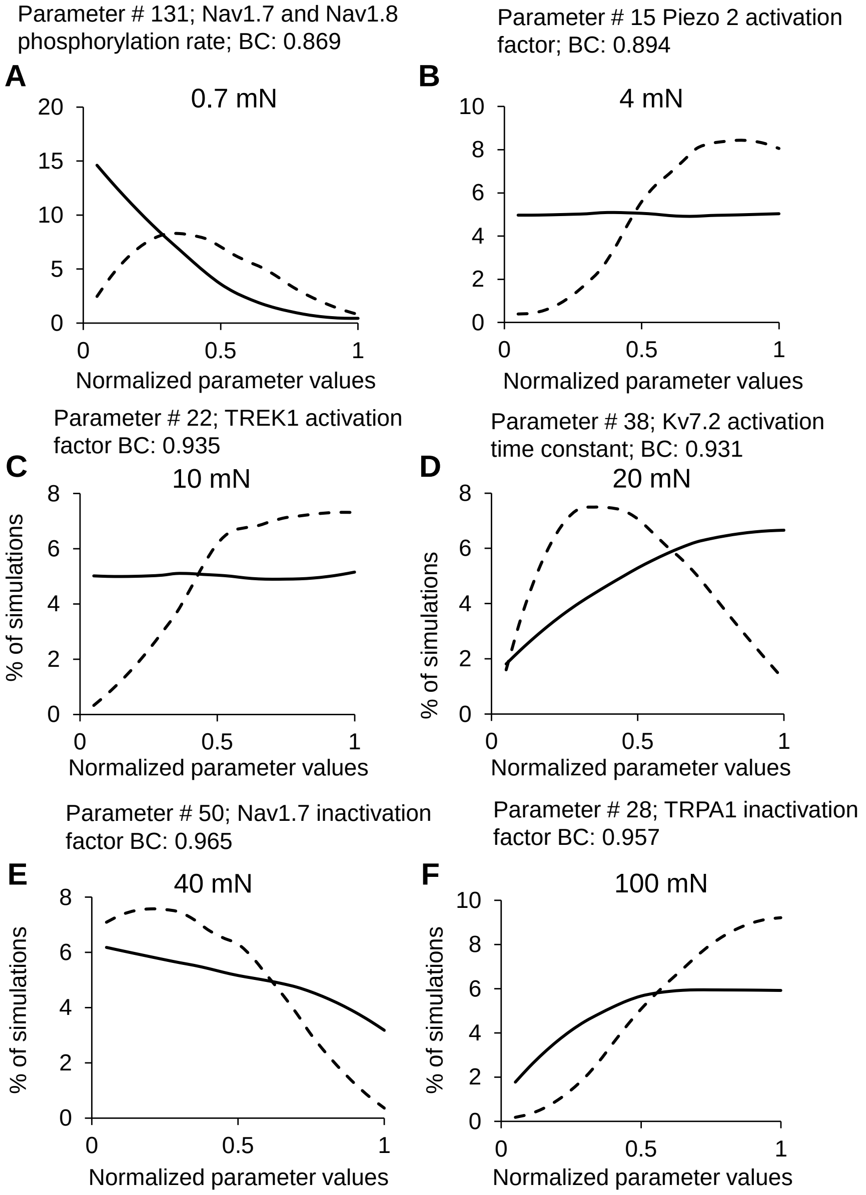

Figure S3. Distributions of the values of parameters that demonstrated the lowest Bhattacharyya coefficients (BC) across the sensitized neuron group simulations (solid lines) and the non-sensitized neuron group simulations (dashed lines), where the neuron groups were classified based on the number of APs fired after (A) 0.7 mN, (B) 4 mN, (C) 10 mN, (D) 20 mN, (E) 40 mN, and (F) 100 mN forces were applied as the input. The *x*-axis designates the normalized values of the parameters, and the *y*-axis represents the percentage of simulations in each neuron group in which the parameter values fell within a particular range (described in the "Methods" section). The number of simulations in the sensitized and non-sensitized groups were, respectively, 306 and 6,134 for 0.7 mN; 837 and 13,625 for 4 mN; 1,007 and 13,326 for 10 mN; 529 and 14,484 for 20 mN; 1,319 and 14,311 for 40 mN; and 2,575 and 17,509 for 100 mN of mechanical force.

Table S4. Total number of APs fired before and after the addition of an inflammatory mediator and the associated fold changes for 14 different modifications

	#APs fired before inflammation	#APs fired after inflammation	Fold change
**Knocking out the channel or down-regulating the biochemical processes**			
Nominal model	14	114	8.14
TRPA1 KO	12	59	4.92
Piezo2 KO	6	108	18.00
Kv7.2 KO	53	513	9.68
GPCR phosphorylation	14	106	7.57
$G_{\alpha q}$ activation	14	804	57.43
PKA inactivation	14	174	12.43
Nav1.8 and Nav1.7 phosphorylation	14	169	12.07
**Increasing channel expression or up-regulating the biochemical processes**			
Nominal model	14	114	8.14
TRPA1 2-fold increase	6	12	2.00
Piezo2 2-fold increase	22	126	5.73
Kv7.2 2-fold increase	8	60	7.50
GPCR phosphorylation	14	788	56.29
$G_{\alpha q}$ activation	14	119	8.50
PKA inactivation	14	33	2.36
Nav1.8 and Nav1.7 phosphorylation	14	71	5.07

Model equations for description of transmembrane currents, endoplasmic reticulum (ER) mechanisms, Nernst potentials, and mass balance of intracellular Na^+, K^+, and Ca^{2+} ions

Transmembrane mechanisms

1. Voltage-gated Nav1.8 channel

$$\frac{\mathrm{dNav1.8_m}}{\mathrm{d}t}=\frac{\mathrm{-Nav1.8_m+Nav1.8_{mss}}}{\tau_{\mathrm{m_{Nav1.8}}}}$$

$$\frac{\mathrm{dNav1.8_h}}{\mathrm{d}t}=\frac{\mathrm{-Nav1.8_h+Nav1.8_{hss}}}{\tau_{\mathrm{h_{Nav1.8}}}}$$

$$\mathrm{am_r}=\frac{7.2}{1+e^{((V_\mathrm{m}-0.063)/7.86)}}$$

$$\mathrm{bm_r}=\frac{7.4}{1+e^{((V_\mathrm{m}+53.06)/19.34)}}$$

$$\mathrm{ah_r}=0.003+\frac{1.63}{1+e^{((V_\mathrm{m}+68.5)/10.01)}}$$

$$\mathrm{bh_r}=0.81-\frac{0.81}{1+e^{((V_\mathrm{m}-11.44)/13.12)}}$$

$$\tau_{\mathrm{m_{Nav1.8}}}=\frac{1}{\mathrm{am_r+bm_r}}$$

$$\tau_{\mathrm{h_{Nav1.8}}}=\frac{1}{\mathrm{ah_r+bh_r}}$$

$$\mathrm{Nav1.8_{mss}}=\frac{1}{1+e^{\left(\frac{\mathrm{Vm_{Nav1.8}}-V_\mathrm{m}}{\mathrm{kact_{Nav1.8}}}\right)}}$$

$$\mathrm{Nav1.8_{hss}}=\frac{1}{1+e^{\left(\frac{V_\mathrm{m}+\mathrm{Vh_{Nav1.8}}}{\mathrm{kinact_{Nav1.8}}}\right)}}$$

$$I_{\mathrm{Nav1.8}}=\mathrm{Imax_{Nav1.8}}\cdot\mathrm{Nav1.8_m^2}\cdot\mathrm{Nav1.8_h}\cdot(V_\mathrm{m}-V_\mathrm{Na})$$

2. Voltage-gated Nav1.9 channel

$$\frac{\mathrm{dNav1.9_m}}{\mathrm{d}t}=\frac{\mathrm{-Nav1.9_m+Nav1.9_{mss}}}{\tau_{\mathrm{m_{Nav1.9}}}}$$

$$\frac{dNav1.9_h}{dt}=\frac{-Nav1.9_h+Nav1.9_{hss}}{\tau_{h_{Nav1.9}}}$$

$$am_{1.9}=\frac{1.548}{1+e^{((V_m-11.01)/-14.871)}}$$

$$bm_{1.9}=\frac{8.685}{1+e^{((V_m+112.4)/22.9)}}$$

$$ah_{1.9}=\frac{0.2574}{1+e^{((V_m+63.264)/3.719)}}$$

$$bh_{1.9}=\frac{0.54}{1+e^{((V_m+0.28)/-0.093)}}$$

$$\tau_{m_{Nav1.9}}=\frac{1}{am_{1.9}+bm_{1.9}}$$

$$\tau_{h_{Nav1.9}}=\frac{1}{ah_{1.9}+bh_{1.9}}$$

$$Nav1.9_{mss}=\frac{am_{1.9}}{am_{1.9}+bm_{1.9}}$$

$$Nav1.9_{hss}=\frac{ah_{1.9}}{ah_{1.9}+bh_{1.9}}$$

$$I_{Nav1.9}= Imax_{Nav1.9}\cdot Nav1.9_m^2\cdot Nav1.9_h\cdot(V_m-V_{Na})$$

3. Voltage-gated Nav1.7 channel

$$\frac{dNav1.7_m}{dt}=\frac{-Nav1.7_m+Nav1.7_{mss}}{\tau_{m_{Nav1.7}}}$$

$$\frac{dNav1.7_h}{dt}=\frac{-Nav1.7_h+Nav1.7_{hss}}{\tau_{h_{Nav1.7}}}$$

$$am_{1.7}=\frac{15.5}{1+e^{((V_m-5)/-12.08)}}$$

$$bm_{1.7}=\frac{35.2}{1+e^{((V_m+72.7)/16.7)}}$$

$$ah_{1.7}=0.24\cdot e^{(-\frac{V_m+115}{46.33})}$$

$$bh_{1.7}=4.32\cdot(1+e^{(\frac{V_m-11.8}{-12})})$$

$$\tau m_{Nav1.7} = \frac{1}{am_{1.7} + bm_{1.7}}$$

$$\tau h_{Nav1.7} = \frac{1}{ah_{1.7} + bh_{1.7}}$$

$$Nav1.7_{mss} = \frac{1}{1 + e^{(\frac{Vm_{Nav1.7} - V_m}{kact_{Nav1.7}})}}$$

$$Nav1.7_{hss} = \frac{1}{1 + e^{(\frac{V_m + Vh_{Nav1.7}}{kinact_{Nav1.7}})}}$$

$$I_{Nav1.7} = Imax_{Nav1.7} \cdot Nav1.7_m^2 \cdot Nav1.7_h \cdot (V_m - V_{Na})$$

4. Mechanosensitive Piezo2 channel

$$\frac{dPiezo_m}{dt} = \frac{Piezo_m + Piezo_{mss}}{\tau act_{Piezo}}$$

$$\frac{dPiezo_h}{dt} = \frac{Piezo_h + Piezo_{hss}}{\tau inact_{Piezo}}$$

$$Piezo_{mss} = \frac{1}{1 + e^{(\frac{Vm_{Piezo} - Mechforce}{kact_{Piezo}})}}$$

$$Piezo_{hss} = 1 - \frac{1}{1 + e^{(\frac{Vh_{Piezo} - Mechforce}{kinact_{Piezo}})}}$$

$$I_{PiezoNa} = Imax_{Piezo} \cdot Piezo_m^4 \cdot Piezo_h^2 \cdot (V_m - V_{Na})$$

$$I_{PiezoCa} = Imax_{Piezo} \cdot Piezo_m^4 \cdot Piezo_h^2 \cdot (V_m - V_{Ca})$$

$$I_{Piezo} = I_{PiezoNa} + I_{PiezoCa}$$

5. Mechanosensitive TRPA1 channel

$$\frac{dTRPA1_m}{dt} = \frac{-TRPA1_m + TRPA1_{mss}}{\tau act_{TRPA1}}$$

$$\frac{dTRPA1_h}{dt} = \frac{-TRPA1_h + TRPA1_{hss}}{\tau inact_{TRPA1}}$$

$$TRPA1_{mss} = \frac{1}{1 + e^{(\frac{Vm_{TRPA1} - Mechforce}{kact_{TRPA1}})}}$$

$$TRPA1_{hss}=1-\frac{1}{1+e^{(\frac{Vh_{TRPA1}-Mechforce}{kinact_{TRPA1}})}}$$

$$I_{TRPA1}= Imax_{TRPA1}\cdot TRPA1_m^2\cdot TRPA1_h\ \cdot(V_m-V_{Na})$$

6. Mechanosensitive two-pore TREK-1 channel

$$\frac{dTREK_m}{dt}=\frac{-TREK_m+TREK_{mss}}{\tau act_{TREK}}$$

$$TREK_{mss}=\frac{1}{1+e^{(\frac{Vm_{TREK}-Mechforce}{kact_{TREK}})}}$$

$$I_{TREK1}= Imax_{TREK}\cdot TREK_m^2\ \cdot(V_m-V_K)$$

Mechforce = 0.7, 4, 10, 20, 40, or 100 mN

7. pH-mediated ASIC3 channel

$$\frac{dASIC3_m}{dt}=\frac{-ASIC3_m+ASIC3_{mss}}{\tau act_{ASIC3}}$$

$$\frac{dASIC3_h}{dt}=\frac{-ASIC3_h+ASIC3_{hss}}{\tau inact_{ASIC3}}$$

$$\tau inact_{ASIC3}=197.36\ \cdot pH^2 - 1738.9\ \cdot pH + 3968.1$$

$$ASIC3_{mss}=\frac{1}{1+e^{(\frac{Vm_{ASIC3}-pH}{kact_{ASIC3}})}}$$

$$ASIC3_{hss}=1-\frac{1}{1+e^{(\frac{Vh_{ASIC3}-pH}{kinact_{ASIC3}})}}$$

$$I_{ASIC3}= Imax_{ASIC3}\cdot ASIC3_m\cdot ASIC3_h\ \cdot(V_m-V_{Na})$$

pH = 7.5

8. Voltage-gated Kv7.2 channel

$$\frac{dKv7_n}{dt}=\frac{-Kv7_n+Kv7_{nss}}{\tau_{n_{Kv7}}}$$

$$an_{Kv7}=k1act_{Kv7}\cdot e^{(\frac{V_m+Vm_{Kv7}}{k2act_{Kv7}})}$$

$$bn_{Kv7}=k1inact_{Kv7}\cdot e^{-(\frac{V_m+Vm_{Kv7}}{k2inact_{Kv7}})}$$

$$\tau_{n_{Kv7}}=\frac{1}{an_{Kv7}+bn_{Kv7}}$$

$$Kv7_{nss}=\frac{1}{1+e^{(\frac{-V_m-Vm_{Kv7}}{\tau act_{Kv7}})}}$$

$$I_{Kv7.2}= Imax_{Kv7}\cdot Kv7_n^2\cdot(V_m-V_K)$$

9. Delayed-rectifier Kv1.1 K⁺ channel

$$\frac{dKv1.1_n}{dt}=\frac{-Kv1.1_n+Kv1.1_{nss}}{\tau act_{Kv1.1}}$$

$$Kv1.1_{nss}=\frac{\delta_{Kv1.1}}{1+e^{(\frac{Vm_{Kv1.1}-V_m}{kact_{Kv1.1}})}}$$

$$I_{Kv1.1}= Imax_{Kv1.1}\cdot Kv1.1_n^2\cdot(V_m-V_K)$$

10. Voltage-gated A-type K⁺ channel

$$\frac{dKa_n}{dt}=\frac{-Ka_n+Ka_{nss}}{\tau_{n_{Ka}}}$$

$$\frac{dKa_{hfast}}{dt}=\frac{-Ka_{hfast}+Ka_{hfastss}}{\tau_{hfast_{Ka}}}$$

$$\frac{dKa_{hslow}}{dt}=\frac{-Ka_{hslow}+Ka_{hslowss}}{\tau_{hslow_{Ka}}}$$

$$Ka_{nss}=\frac{1}{1+e^{(\frac{-V_m-40.8}{9.5})}}$$

$$Ka_{hfastss}=\frac{1}{1+e^{(\frac{V_m+74.2}{9.6})}}$$

$$\tau_{n_{Ka}}= 1.2 + 2.56\cdot e^{(-2\cdot\left(\frac{V_m+60}{45.768}\right)^2)}$$

$$\tau_{hfast_{Ka}}= 25.46 + 67.41\cdot e^{(-2\cdot\left(\frac{V_m+50}{21.95}\right)^2)}$$

$$\tau_{hslow_{Ka}}= 200 + 587.4\cdot e^{(-\left(\frac{V_m}{47.77}\right)^2)}$$

I_{Ka}= $Imax_{Ka} \cdot Ka_n \cdot (0.3Ka_{hfast}+0.7Ka_{hslow}) \cdot (V_m - V_K)$

11. Large-conductance Ca^{2+}-activated K^+ channel

$$\frac{dBKCa_n}{dt} = \frac{-BKCa_n + BKCa_{nss}}{\tau_{nBKCa}}$$

p_{Ca}= $\log_{10} \cdot (Ca_i e^{-3})$

$kact_{BKCa}$= $(-43.4 \cdot p_{Ca})-203$

sf_{BKCa}= $33.88 \cdot e^{-(p_{Ca}+5.42)/1.85^2}$

$$BKCa_{nss} = \frac{1}{1+e^{(\frac{kact_{BKCa}-V_m}{sf_{BKCa}})}}$$

$$\tau_{nBKCa} = 5.55 \cdot e^{\frac{V_m}{42.91}} + 0.75 - (0.12 \cdot V_m)$$

I_{BKCa}= $Imax_{BKCa} \cdot BKCa_n^2 \cdot (V_m - V_K)$

12. T-type voltage-gated Ca^{2+} channel

$$\frac{dCaT_m}{dt} = \frac{-CaT_m + CaT_{mss}}{\tau act_{CaT}}$$

$$\frac{dCaT_h}{dt} = \frac{-CaT_h + CaT_{hss}}{\tau inact_{CaT}}$$

$$CaT_{mss} = \frac{1}{1+e^{(\frac{V_m - Vm_{CaT}}{kact_{CaT}})}}$$

$$CaT_{hss} = 1 - \frac{1}{1+e^{(\frac{V_m - Vh_{CaT}}{kinact_{CaT}})}}$$

I_{CaT}= $Imax_{CaT} \cdot CaT_m^2 \cdot CaT_h \cdot (V_m - V_{Ca})$

13. L-type voltage-gated Ca^{2+} channel

$$\frac{dCaL_m}{dt} = \frac{-CaL_m + CaL_{mss}}{\tau act_{CaL}}$$

$$\frac{dCaL_h}{dt} = \frac{-CaL_h + CaL_{hss}}{\tau inact_{CaL}}$$

$$\mathrm{CaL_{mss}} = \frac{1}{1+e^{\left(\frac{\mathrm{Vm_{CaL}} - V_m}{\mathrm{kact_{CaL}}}\right)}}$$

$$\mathrm{CaL_{hss}} = 1 - \frac{1}{1+e^{\left(\frac{\mathrm{Vh_{CaL}} - V_m}{\mathrm{kinact_{CaL}}}\right)}}$$

$$\mathrm{hCa_{CaL}} = \frac{1}{1+(\mathrm{Ca_i}/1e^{-6})^4}$$

$$I_{\mathrm{CaL}} = \mathrm{Imax_{CaL}} \cdot \mathrm{CaL_m} \cdot \mathrm{CaL_h} \cdot \mathrm{hCa_{CaL}} \cdot (V_m - V_{\mathrm{Ca}})$$

14. NaK pump

$$I_{\mathrm{NaK}} = \mathrm{Imax_{NaK}} \cdot \frac{\mathrm{K_o^2}}{\mathrm{K_o^2} + \mathrm{KK_{NaK}^2}} \cdot \frac{\mathrm{Na_i^{nH_{Na}}}}{\mathrm{Na_i^{nH_{Na}}} + \mathrm{KNa_{NaK}^{nH_{Na}}}} \cdot \frac{V_m + 70}{V_m + 180}$$

15. PMCA pump

$$I_{\mathrm{PMCA}} = \mathrm{Imax_{PMCA}} \cdot \frac{\mathrm{Ca_i}}{\mathrm{Ca_i} + \mathrm{KCa_{PMCA}}}$$

16. Na^+-Ca^{2+} exchanger

$$\mathrm{kqa} = e^{\frac{0.35 \cdot V_m}{k_{\mathrm{NCX}}}}$$

$$\mathrm{kb_{NCX}} = e^{\frac{-0.65 \cdot V_m}{k_{\mathrm{NCX}}}}$$

$$I_{\mathrm{NCX}} = \mathrm{Imax_{NCX}} \cdot (\mathrm{kqa} \cdot \mathrm{Na_i^3} \cdot \mathrm{Ca_o}) - \frac{\mathrm{kb_{NCX}} \cdot \mathrm{Ca_i} \cdot \mathrm{Na_o^3}}{(\mathrm{kNa^3} + \mathrm{Na_o^3}) \cdot (\mathrm{kCa} + \mathrm{Ca_o}) \cdot (1 + 0.1\mathrm{kb_{NCX}})}$$

17. Passive K^+ leak channel

$$I_{\mathrm{Kleak}} = \mathrm{Imax_{Kleak}} \cdot (V_m - (-45))$$

ER mechanisms

1. IP_3 receptor flux

$$\frac{\mathrm{dhIP_3}}{\mathrm{d}t} = \mathrm{kf_{IP3}} \cdot (\mathrm{kb_{IP3}} - (\mathrm{Ca_i} + \mathrm{kb_{IP3}}) \cdot \mathrm{hIP_3})$$

$$I_{IP3R} = Imax_{IP3R} \cdot \left(\frac{IP_3}{IP_3 + k_{IP3}}\right) \cdot \left(\frac{Ca_i}{Ca_i + kCa_{IP3}} \cdot hIP_3\right)^3 \cdot \left(1 - \frac{Ca_i}{Ca_{ER}}\right)$$

2. SERCA pump

$$I_{SERCA} = Imax_{SERCA} \cdot \left(\frac{Ca_i^2}{Ca_i^2 + KCa_{SERCA}^2}\right)$$

3. ER leak current

$$I_{leakER} = 5\times10^{-7} \cdot \left(1 - \frac{Ca_i}{Ca_{ER}}\right)$$

$$I_{leakER} = Imax_{ERleak} \cdot \left(1 - \frac{Ca_i}{Ca_{ER}}\right) \qquad \text{if } Ca_i > KTCa$$

4. Ryanodine receptor flux

$$I_{CICR} = Imax_{CICR} \cdot \left(\frac{Ca_i}{Ca_i + KCa_{CICR}}\right) \cdot (Ca_{ER} - Ca_i) \qquad \text{if } Ca_i > KTCa$$

$$I_{CICR} = 0$$

5. Ca_i^{2+} buffering in cytosol and ER

$$\beta_{ER} = \frac{CSQN \cdot K_{CSQN}}{(K_{CSQN} + Ca_{ER})^2}$$

6. PKC activation and signaling

$$\frac{d[RG_s]}{dt} = k_{rG} \cdot epsilon_G \cdot [RTG] - \left(k_{rG} + \frac{k_{pG} \cdot [PGE_2]}{(K_{1G} + [PGE_2])}\right) \cdot [RG_s] - k_{rG}[RPG_s]$$

$$\frac{d[RPG_s]}{dt} = [PGE_2] \cdot \left(\frac{k_{pG} \cdot [RG_s]}{(K_{1G} + [PGE_2])}\right) \cdot \left(\frac{k_{eG} \cdot [RPG_s]}{(K_{2G} + [PGE_2])}\right)$$

$$\rho_{rG} = \alpha_G \left(\frac{[PGE_2] \cdot [RG_s]}{epsilon_G \cdot [RTG] \cdot (K_{1G} + [PGE_2])}\right)$$

$$\frac{d[G_{\alpha q}]}{dt} = k_a(\delta + \rho_{rG})([G_T] - [G_{\alpha q}]) - k_d[G_{\alpha q}]$$

$$r_{hG}=\alpha_G\left(\frac{[Ca^{2+}]_i}{[Ca^{2+}]_i+k_{cG}}\right)G$$

$$\frac{d[PIP_2]}{dt}= -(r_{hG}+r_{rG})[PIP_2]-r_{rG}\cdot\Upsilon_G[IP_3]+r_{rG}[PIP_{2T}]$$

$$\frac{d[IP_3]}{dt}= \frac{r_{hG}}{\Upsilon_G}[PIP_2]-k_{degG}[IP_3]$$

$$\frac{d[PLC_{inact}]}{dt}= -(k_{PLCinact})[PLC_{act}]-k_{PLCact}\cdot[PLC_{inact3}]\cdot[G_{\alpha q}]$$

$$\frac{d[PLC_{act}]}{dt}= k_{PLCact}\cdot[PLC_{inact3}]\cdot[G_{\alpha q}]-(k_{PLCinact})[PLC_{act}]$$

$$\frac{dDAG]}{dt}= k_{hyd}\cdot[PIP_2]\cdot[PLC_{act}]-k_{deg}[DAG]-kact_{PKC}[DAG]\cdot[PKC_{inact}] + (k_{inactPKC})[PKC_{act}] + k_{off}[DAG\_PKC]$$

$$\frac{d[PKC_{inact}]}{dt}= -k_{actPKC}\cdot[PKC_{inact}]\cdot[DAG] + k_{inactPKC}\ [PKC_{act}] + k_{off}[DAG\_PKC]$$

$$\frac{dDAG\_PKC]}{dt}= k_{dp}[PKC_{act}] - k_{off}[DAG\_PKC]$$

$$\frac{d[PKC_{act}]}{dt}= k_{actPKC}\cdot[PKC_{inact}]\cdot[DAG] - k_{inactPKC}\ [PKC_{act}]- k_{dp}[PKC_{act}]$$

7. PKA activation and signaling

$$GPCR_{act}=\alpha_G\left(\frac{PGE_2\cdot[GPCR_{tot}]}{PGE_2+k_{EPdiss}}\right)$$

$$\frac{d[G_{\alpha s}]}{dt}=k1_{G\alpha s}[GPCR_{act}]\cdot\left(\frac{[G_{\alpha\beta\gamma}]}{(K_{EPdiss}+[G_{\alpha\beta\gamma}]}\right)-k2_{G\alpha s}[G_{\alpha s}]$$

$$[G\alpha s_{inact}]=[G\alpha s_{tot}]-[G_{\alpha s}]-[G_{\alpha\beta\gamma}]$$

$$\frac{d[G_{\alpha\beta\gamma}]}{dt}= -k1_{G\alpha s}[GPCR_{act}]\cdot\left(\frac{[G_{\alpha\beta\gamma}]}{K_{EPdiss}+[G_{\alpha\beta\gamma}]}\right)+ k3_{G\alpha s}\left[G_{\beta\gamma}\right][G_{\alpha s}] - k4_{G\alpha s}\left[G_{\alpha\beta\gamma}\right]$$

$$\frac{d[G_{\beta\gamma}]}{dt}=k1_{G\alpha s}[GPCR_{act}]\cdot\left(\frac{[G_{\alpha\beta\gamma}]}{K_{EPdiss}+[G_{\alpha\beta\gamma}]}\right)- k3_{G\alpha s}\left[G_{\beta\gamma}\right][G_{\alpha s}] + k4_{G\alpha s}\left[G_{\alpha\beta\gamma}\right]$$

$$[AC_{act}]=\frac{[AC_{tot}]\cdot[G_{\alpha s}]}{[G_{\alpha s}]+kAC_{diss}}$$

$$[AC_{inact}]=[AC_{tot}]-[AC_{act}]$$

$$\frac{d[cAMP]}{dt}=\left(\frac{k5_{G\alpha s}[AC_{act}]}{kAC_{\beta\gamma diss}+[G_{\beta\gamma}]}\right)+k6_{G\alpha s}[AC_{act}]-k_{degcAMP}[cAMP]$$

$$\frac{d[RC]}{dt}=-k_{fPKA}[RC]\cdot[cAMP]^2+k_{bPKA}[RC_{cAMP2}]$$

$$\frac{d[RC_{cAMP2}]}{dt}=k_{fPKA}[RC]\cdot[cAMP]^2-k_{bPKA}[RC_{cAMP2}]-k_{f9}[RC_{cAMP2}]\cdot[cAMP]^2+k_{b9}[RC_{cAMP4}]$$

$$\frac{d[RC_{cAMP4}]}{dt}=k_{f9}[RC_{cAMP2}]\cdot[cAMP]^2-k_{b9}[RC_{cAMP4}]-k_{f10}[RC_{cAMP4}]+k_{b10}[RC_{cAMP}]\cdot[PKA]^2$$

$$\frac{d[RC_{cAMP}]}{dt}=k_{f10}[RC_{cAMP4}]-k_{b10}[RC_{cAMP}]\cdot[PKA]^2$$

$$\frac{d[PKA]}{dt}=2k_{f10}[RC_{cAMP4}]-k_{b10}[RC_{cAMP}]\cdot[PKA]^2$$

8. Equations for sensitization of nociceptor

First, we used the two equations shown below to compute the magnitude of change (ΔV_m for Nav1.7, Nav1.8, and Kv1.1, and ΔMech for TRPA1) induced by PKC and PKA:

$$\Delta V_{mPKC} \text{ or } \Delta Mech_{PKC}=\frac{15}{1+e^{\frac{khalf_{PKC}-[PKC]}{kslope_{PKC}}}}$$

$$\Delta V_{mPKA} \text{ or } \Delta Mech_{PKA}=\frac{13}{1+e^{\frac{khalf_{PKA}-[PKA]}{kslope_{PKA}}}}$$

where [PKC] and [PKA] denote the instantaneous concentrations of PKC and PKA (zero in the absence of an inflammatory mediator), respectively, and $khalf_{PKC}$, $khalf_{PKA}$, $kslope_{PKC}$, and $kslope_{PKA}$ denote the phosphorylation and steepness factors for PKC and PKA (Nicol et al., 1997; Wu et al., 2012). Next, we computed the new values of the activation and inactivation thresholds for each of the four ion channels using the following equations:

$$\left.\begin{aligned} Vact_{new\_i} &= Vact_i-\Delta V_{mPKC}-\Delta V_{mPKA} \\ Vinact_{new\_i} &= Vinact_i+\Delta V_{mPKC}+\Delta V_{mPKA} \end{aligned}\right\} \text{Inflammatory mediator} > 0$$

$$\left.\begin{aligned} Vact_{new\_i} &= Vact_i+\Delta V_{mPKC}+\Delta V_{mPKA} \\ Vinact_{new\_i} &= Vinact_i-\Delta V_{mPKC}-\Delta V_{mPKA} \end{aligned}\right\} \text{Inflammatory mediator} = 0$$

where i denotes one of the four channels, i.e., TRPA1, Nav1.7, Nav1.8, and Kv1.1, $Vact_{new\_i}$ and $Vinact_{new\_i}$ denote the new values, and $Vact_i$ and $Vinact_i$ denote the nominal values, respectively, of the activation and inactivation thresholds for each of the four channels.

9. ODEs for change in the activation and inactivation variables of Nav1.7, Nav1.8, Kv1.1, and TRPA1

$$\frac{\mathrm{d}[V\mathrm{hact}_{\mathrm{TTXs}}]}{\mathrm{d}t}=\left(\frac{V\mathrm{act}_{\mathrm{new}}\text{-}V\mathrm{hact}_{\mathrm{TTXs}}}{\tau_{\mathrm{Navphos}}}\right)$$

$$\frac{\mathrm{d}[V\mathrm{hinact}_{\mathrm{TTXs}}]}{\mathrm{d}t}=\left(\frac{V\mathrm{inact}_{\mathrm{new}}\text{-}V\mathrm{hinact}_{\mathrm{TTXs}}}{\tau_{\mathrm{Navphos}}}\right)$$

$$\frac{\mathrm{d}[V\mathrm{hact}_{\mathrm{TTXr}}]}{\mathrm{d}t}=\left(\frac{V\mathrm{act}_{\mathrm{new}}\text{-}V\mathrm{hact}_{\mathrm{TTXr}}}{\tau_{\mathrm{Navphos}}}\right)$$

$$\frac{\mathrm{d}[V\mathrm{hinact}_{\mathrm{TTXr}}]}{\mathrm{d}t}=\left(\frac{V\mathrm{inact}_{\mathrm{new}}\text{-}V\mathrm{hinact}_{\mathrm{TTXr}}}{\tau_{\mathrm{Navphos}}}\right)$$

$$\frac{\mathrm{d}[V\mathrm{hact}_{\mathrm{Kv1.1}}]}{\mathrm{d}t}=\left(\frac{V\mathrm{act}_{\mathrm{new}}\text{-}V\mathrm{hact}_{\mathrm{Kv1.1}}}{\tau_{\mathrm{Kv1.1phos}}}\right)$$

$$\frac{\mathrm{d}[TRPA1_{aM}]}{\mathrm{d}t}=\left(\frac{V\mathrm{act}_{\mathrm{new}}\text{-}TRPA1_{aM}}{\tau_{\mathrm{TRPA1phos}}}\right)$$

Nernst potential calculations

$$V_{\mathrm{Na}}=\frac{\mathrm{R\cdot T}}{\mathrm{zNa\cdot F}}\cdot\log\left(\frac{\mathrm{Na_o}}{\mathrm{Na_i}}\right)$$
$$V_{\mathrm{K}}=\frac{\mathrm{R\cdot T}}{\mathrm{zK\cdot F}}\cdot\log\left(\frac{\mathrm{K_o}}{\mathrm{K_i}}\right)$$
$$V_{\mathrm{Ca}}=\frac{\mathrm{R\cdot T}}{\mathrm{zCa\cdot F}}\cdot\log\left(\frac{\mathrm{Ca_o}}{\mathrm{Ca_i}}\right)$$

Ionic balances

$$\frac{\mathrm{dCa_i}}{\mathrm{d}t}=\text{-}(I_{\mathrm{CaT}}+I_{\mathrm{CaL}}+I_{\mathrm{PMCA}}-2I_{\mathrm{NCX}}+I_{\mathrm{PiezoCa}})/(\mathrm{zCa\cdot F\cdot 0.7vol}))\text{-}I_{\mathrm{SERCA}}\text{-}I_{\mathrm{leakER}}\text{-}I_{\mathrm{IP3}}\text{-}I_{\mathrm{CICR}})/(1/(1+370))$$

$$\frac{\mathrm{dCa_{ER}}}{\mathrm{d}t}=I_{\mathrm{SERCA}}\text{-}I_{\mathrm{leakER}}\text{-}I_{\mathrm{IP3}}\text{-}I_{\mathrm{CICR}}/\beta_{ER}$$

$$\frac{\mathrm{dNa_i}}{\mathrm{d}t}=\text{-}(I_{\mathrm{Nav1.8}}+I_{\mathrm{Nav1.9}}+I_{\mathrm{Nav1.7}}+I_{\mathrm{PiezoNa}}+I_{\mathrm{TRPA1}}+3I_{\mathrm{NaK}}+3I_{\mathrm{NCX}})/(\mathrm{zNa\cdot F\cdot vol})$$

$$\frac{\mathrm{dK_i}}{\mathrm{d}t}=\text{-}(I_{\mathrm{TREK1}}+I_{\mathrm{Kv7.2}}+I_{\mathrm{Kv1.1}}+I_{\mathrm{BKCa}}+I_{\mathrm{Ka}}+I_{\mathrm{Kleak}}\text{-}2I_{\mathrm{NaK}})/(\mathrm{zK\cdot F\cdot vol})$$

References

Bennett, M.R., Farnell, L., and Gibson, W.G. (2005). A quantitative description of the contraction of blood vessels following the release of noradrenaline from sympathetic varicosities. *J Theor Biol* 234(1)**,** 107-122. doi: 10.1016/j.jtbi.2004.11.013.

Gold, M.S., and Flake, N.M. (2005). Inflammation-mediated hyperexcitability of sensory neurons. *Neurosignals* 14(4)**,** 147-157. doi: 10.1159/000087653.

Kapela, A., Bezerianos, A., and Tsoukias, N.M. (2008). A mathematical model of Ca^{2+} dynamics in rat mesenteric smooth muscle cell: agonist and NO stimulation. *J Theor Biol* 253(2)**,** 238-260. doi: 10.1016/j.jtbi.2008.03.004.

Leander, R., and Friedman, A. (2014). Modulation of the cAMP response by Gαi and Gβγ: a computational study of G protein signaling in immune cells. *Bull Math Biol* 76(6)**,** 1352-1375. doi: 10.1007/s11538-014-9964-4.

Li, Z.H., Cui, D., Qiu, C.J., and Song, X.J. (2019). Cyclic nucleotide signaling in sensory neuron hyperexcitability and chronic pain after nerve injury. *Neurobiol Pain* 6**,** 100028. doi: 10.1016/j.ynpai.2019.100028.

Lindskog, M., Kim, M., Wikstrom, M.A., Blackwell, K.T., and Kotaleski, J.H. (2006). Transient calcium and dopamine increase PKA activity and DARPP-32 phosphorylation. *PLoS Comput Biol* 2(9)**,** e119. doi: 10.1371/journal.pcbi.0020119.

Mohan, K., Nosbisch, J.L., Elston, T.C., Bear, J.E., and Haugh, J.M. (2017). A reaction-diffusion model explains amplification of the PLC/PKC pathway in fibroblast chemotaxis. *Biophys J* 113(1)**,** 185-194. doi: 10.1016/j.bpj.2017.05.035.

Nagaraja, S., Queme, L.F., Hofmann, M.C., Tewari, S.G., Jankowski, M.P., and Reifman, J. (2021). *In silico* identification of key factors driving the response of muscle sensory neurons to noxious stimuli. *Front Neurosci* 15**,** 719735. doi: 10.3389/fnins.2021.719735.

Nicol, G.D., Vasko, M.R., and Evans, A.R. (1997). Prostaglandins suppress an outward potassium current in embryonic rat sensory neurons. *J Neurophysiol* 77(1)**,** 167-176. doi: 10.1152/jn.1997.77.1.167.

Wu, D.F., Chandra, D., McMahon, T., Wang, D., Dadgar, J., Kharazia, V.N., et al. (2012). PKCε phosphorylation of the sodium channel Nav1.8 increases channel function and produces mechanical hyperalgesia in mice. *J Clin Invest* 122(4)**,** 1306-1315. doi: 10.1172/JCI61934.
